# Computational Literature-based Discovery for Natural Products Research: Current State and Future Prospects

**DOI:** 10.3389/fbinf.2022.827207

**Published:** 2022-03-15

**Authors:** Andreas Lardos, Ahmad Aghaebrahimian, Anna Koroleva, Julia Sidorova, Evelyn Wolfram, Maria Anisimova, Manuel Gil

**Affiliations:** ^1^ Natural Product Chemistry and Phytopharmacy Research Group, Institute of Chemistry and Biotechnology, School of Life Sciences and Facility Management, Zurich University of Applied Sciences (ZHAW), Waedenswil, Switzerland; ^2^ Institute of Applied Simulation, School of Life Sciences and Facility Management, Zürich University of Applied Sciences (ZHAW), Waedenswil, Switzerland; ^3^ Swiss Institute of Bioinformatics (SIB), Lausanne, Switzerland; ^4^ Instituto de Tecnología del Conocimiento, Universidad Complutense de Madrid, Madrid, Spain

**Keywords:** literature-based discovery, natural products, text mining, knowledge graph, natural language processing, swanson, semantic integration, ontology

## Abstract

Literature-based discovery (LBD) mines existing literature in order to generate new hypotheses by finding links between previously disconnected pieces of knowledge. Although automated LBD systems are becoming widespread and indispensable in a wide variety of knowledge domains, little has been done to introduce LBD to the field of natural products research. Despite growing knowledge in the natural product domain, most of the accumulated information is found in detached data pools. LBD can facilitate better contextualization and exploitation of this wealth of data, for example by formulating new hypotheses for natural product research, especially in the context of drug discovery and development. Moreover, automated LBD systems promise to accelerate the currently tedious and expensive process of lead identification, optimization, and development. Focusing on natural product research, we briefly reflect the development of automated LBD and summarize its methods and principal data sources. In a thorough review of published use cases of LBD in the biomedical domain, we highlight the immense potential of this data mining approach for natural product research, especially in context with drug discovery or repurposing, mode of action, as well as drug or substance interactions. Most of the 91 natural product-related discoveries in our sample of reported use cases of LBD were addressed at a computer science audience. Therefore, it is the wider goal of this review to introduce automated LBD to researchers who work with natural products and to facilitate the dialogue between this community and the developers of automated LBD systems.

## 1 Introduction

Literature-based discovery (LBD) is a form of knowledge extraction. It aims at discovering new knowledge that is implicitly present in scientific literature. For instance, two concepts A and C that do not occur in the same document or article can still be connected via some other terms B, which can imply a meaningful relation between A and C. A classic example is a discovery made by Don Swanson in pioneering work in the 1980s (Swanson, 1986). He found an article showing that fish oil (A) can reduce vascular reactivity (B), and a different one showing that a reduction in vascular reactivity (B) can treat Raynaud’s syndrome (C). Inspired by this finding, he hypothesised that fish oil (A) can treat Raynaud’s syndrome (C), which was later clinically corroborated.

LBD can be automated. Current LBD systems commonly use databases that store information extracted from scientific literature in the form of semantic predications (subject-predicate-object triples). An informal example is *<fish_oil, reduces, vascular_reactivity>.* There are Natural Language Processing (NLP) methods for extracting these triples. A frequently used database in LBD is SemMedDB (Kilicoglu et al., 2012), created with the information extraction system SemRep (Rindflesch et al., 2005).

LBD has become widespread, but little has been done to introduce it to the field of natural product research. None of the 91 discoveries identified in our sample of LBD use cases (see below) has been published in a natural product-related journal. Similarly, our query for articles in frontiers (https://www.frontiersin.org; conducted on 15 November 2021) using the search term “natural products” in combination with the terms “literature-based discovery”, “natural language processing”, “text mining”, and or “knowledge graph” did not return any results. Focusing on the biomedical domain, the primary aim of this review is to provide an overview of published use cases of LBD linked with natural products. Further, this review introduces automated LBD to researchers working with natural products in general, and especially those focussing on small molecules, secondary metabolites, or herbal multicomponent mixtures. Ultimately, we hope that this work will also contribute to the dialogue between the natural product community and the developers of automated LBD systems.

Natural products can be broadly defined as any substance produced by Nature. Depending on the field of interest, various definitions exist (Chin et al., 2006; Cragg and Pezzuto, 2016; Patridge et al., 2016; Newman and Cragg, 2020). This study is focused on natural products of non-mammalian origin and encompasses, 1) secondary metabolites or small molecules from plants, microorganisms, and higher fungi, including both isolated compounds, and complex mixtures such as herbal extracts or botanicals in general, 2) derivatives of the above group of natural products including also semi-synthetic compounds, 3) animal venoms, 4) minerals and vitamins. This definition largely corresponds to the definition of natural products applied by the Natural Centre for Complementary and Integrative Health (NCCIH, 2020) of the National Health Institute (NIH) (U.S. Department of Health and Human Services, (Natural Products Research—Information for Researchers). Total synthetic compounds, which act as natural product mimics or have a natural product pharmacophore are outside the focus of this study. The reason for neglecting these products is based on the fact that it is often tricky to retrace how these evolved from the initial structural information of some natural product (Newman et al., 2003; Chin et al., 2006). To conduct the required analysis of the history of the corresponding scientific literature is outside the possibilities of this study.

For developing new therapeutic agents, natural products continue to play an important part. Of all new drugs approved worldwide in the past 4 decades, almost 25% are natural products including derivatives or botanical drugs (phytopharmaceuticals) and another 25% are synthetics with a natural product pharmacophore or such acting as natural product mimics (Newman and Cragg, 2020). Historically, plants have been the major source of medicines for humans (Sneader, 2005; Cragg and Pezzuto, 2016) and traditional medicinal systems around the world primarily rely on herbal substances for treating illnesses (Li and Weng, 2017). While roughly 50% of all U.S. Food and Drug Administration (FDA) approved natural product-related drugs by 2013 are plant-derived, the importance of bacteria and fungi as sources for drugs has steadily been increasing since the 1940s (Patridge et al., 2016). Investigation of endogenous microbes in plants or other host organisms, novel microbial metabolites, or animal venoms (Cragg and Pezzuto, 2016) is expanding the range of natural products currently under research.

A key focus in pharmaceutical research during the last decades was the screening of synthetic compound libraries or the investigation of biologicals (Koehn and Carter, 2005). However, a re-emerging interest in drug discovery from natural products is currently taking place (David et al., 2015; Harvey et al., 2015) and the potential for developing innovative drugs from various natural resources is considered remarkable (Koehn and Carter, 2005; Li and Vederas, 2009; Atanasov et al., 2015; Thomford et al., 2018). The plethora and unparalleled diversity of molecules from Nature as well as their evolutionary conditioned optimization for specific biological functions are some of the arguments supporting this view (Ma et al., 2011; Chen et al., 2017).

Regarding the question of how to source and identify potential starting points for natural product drug discovery, there is more than one possibility. Organisms can be sourced directly in their terrestrial or marine habitats by random collection and selection (Li and Vederas, 2009; Henrich and Beutler, 2013; Atanasov et al., 2015), or by exploiting the richness of physical natural product libraries (Brown and Newman, 2006; Chen et al., 2017). A major part of the natural product-derived drugs developed in the past decades, especially those from bacteria, lower fungi, or organisms of marine origin was discovered in this way (Chin et al., 2006). On the other hand, numerous natural products of botanical origin were discovered by studying traditionally used medicinal plants, which is regarded as the ethnopharmacological approach (Fabricant and Farnsworth, 2001; Heinrich, 2010).

In addition to these established approaches in bioprospecting, biological, molecular, and or pharmacological information on the various organisms or their components can be used as starting points for research. In this context, the ecological and the chemosystematic approaches (Ramos, 2012) or the combination of phylogenetic with ethnobotanical information (Saslis-Lagoudakis et al., 2012) is regarded as having a high potential for identifying bioactive compounds. Advances in analytical and computational techniques further expand the range of possibilities, including targeted extraction methods and pre-fractionation, NMR-profiling of extracts, mass-spectroscopy, omic technologies, in particular, metabolomics, and genomics (Harvey et al., 2015; Newman, 2017; Thomford et al., 2018). Computational-experimental approaches such as network pharmacology (Kibble et al., 2015), computer-aided screening of virtual natural product databases (Chen et al., 2017), or various other virtual screening approaches (Atanasov et al., 2015) are being increasingly integrated into drug discovery.

In this review, we argue that LBD is a powerful emerging computational approach for natural product research or in particular drug discovery. Many automated LBD systems exist today, which attempt to take advantage of the wealth of scientific literature, to formulate novel research hypotheses. An immense collection of information on natural products and associated biological entities, such as diseases, genes, receptors, or drugs exists in biomedical literature databases, such as MEDLINE. In addition, and over 120 natural product-specific databases and collections of diverse thematic scopes have been in operation during the last 2 decades (Sorokina and Steinbeck, 2020). Envisaged open database ecosystems offering access to comprehensive and cross-linked metabolomic data on natural products might add a further dimension to future data mining possibilities (Allard et al., 2018).

However, most of the data accumulated in the different scientific fields today is contained in disconnected data pools, which hampers their contextualisation. Automated LBD systems provide an opportunity to contextualize these data by establishing implicit connections between previously non-associated pieces of information. Through evaluation of these hypothetical relationships, relevant discoveries can be made. By manual search, in contrast, the same relationships could only be discovered, if at all, with excessive time expenditure, and tremendous human cognitive effort.

As highlighted above, until now LBD does not appear to have been systematically explored in context with natural products. Therefore, the purpose of reviewing published use cases is to lead the research community to the potential of LBD in this field. We first review the origins, methods, data sources, and systems of automated LBD. We do not aim at an in-depth methodological review, as these already exist (Henry and McInnes, 2017; Gopalakrishnan et al., 2019; Thilakaratne et al., 2019). Rather, we provide an overview of the state of the art. It can function as a starting point for further reading, but the primary aim is to lay a foundation for our review of existing use cases; that is, discoveries made within the LBD paradigm. After a general review in the biomedical domain, we focus on natural products and assess the potential of LBD for future discoveries.

## 2 Review of Automated Literature-Based Discovery

### 2.1 Development of LBD in the Biomedical Domain

Don Swanson (1986) introduced an LBD model known as the ABC paradigm for the first time. The ABC paradigm hypothesizes a meaningful relation between terms A and C if they never occur in the same set of publications, but they both occur together with some terms B in separate publications. The ABC model was later expanded to the AnC model which allows terms A and C to be connected via more than one term (A-B1-…-Bn-C). Discoveries made within the LBD framework are classified into two types known as open and close discovery (Henry and McInnes, 2017; Gopalakrishnan et al., 2019). The goal in the close discovery is to find possible linking term B between two terms, A and C. In the open discovery, however, only one term (term A) is given, and the goal is to find C terms connected to it via various B terms.

The first LBD discoveries were conducted manually, which were highly laborious and time-consuming. Employing automated text mining techniques, Swanson and Smalheiser and Swanson, 1998 proposed the first automated LBD system, called ARROWSMITH, that was faster and more efficient. Automated LBD has started to attract significant interest from the biomedical community, as it assists drug discovery and drug repurposing. For conducting LBD in the biomedical domain, MEDLINE is the principal data source along with other databases of scientific publications such as the Web of Science. The majority of LBD systems use titles and abstracts while few systems explore full texts (Thilakaratne et al., 2019). Terms and relations in different LBD systems can be represented as n-grams, Unified Medical Language System (UMLS) concepts (Rindflesch and Fiszman, 2003; Kilicoglu et al., 2020), Medical Subject Heading (MESH, 2020) terms (Jenssen et al., 2001; Baker et al., 2015; Cameron et al., 2015), or context vectors (Henry and McInnes, 2017).

### 2.2 Major Categories of LBD Systems

There are three major categories of LBD systems known as co-occurrence, semantic, and graph-based systems which we briefly introduce in the following paragraphs. A comprehensive review of the different LBD systems including an overview of available resources is provided by Gopalakrishnan et al. (2019).

Arrowsmith (Smalheiser and Swanson, 1998), DAD (Weeber et al., 2000; Weeber et al., 2001), Litlinker (Pratt and Yetisgen-Yildiz, 2003; Yetisgen-Yildiz and Pratt, 2006), and FACTA^+^ (Tsuruoka et al., 2011) are well-known *co-occurrence* LBD systems. Arrowsmith is the first attempt to automate LBD. It heavily relies on guidance from the user, which influences the search results. The main limitation of Arrowsmith, though, is that it only uses titles, hence, the information present in other parts of an article is not processed. DAD is an interactive LBD support tool for open and close discovery. Representing terms as UMLS concepts and MeSH terms, it has the benefit of being able to merge synonyms and textual variants of a word into one concept. Litlinker incorporates knowledge-based methodologies with a statistical method to find new, potentially causal relations between biomedical terms. Finally, FACTA+ utilizes machine learning for extracting biomedical events based on term co-occurrence.

Co-occurrences of terms do not always imply meaningful relationships and do not allow to specify their exact nature. The second category of LBD systems known as *semantic* systems are proposed to address these issues. A semantic LBD system uses explicit relations between terms that are extracted from texts using NLP systems.

The majority of semantic-based approaches use information extraction systems, such as SemRep, and/or databases such as SemMedDB. Zhang et al. Employing the semantic predications extracted by SemRep, Cohen et al. (2012) introduced an approach called Predication-based Semantic Indexing (PSI) to identify discovery patterns. They do inference through the application of geometric operators in PSI space. Using the relationships in SemMedDB, Zhang et al., 2014b aimed at discovering potential drug-drug interactions via exploring patient clinical data. Utilizing SemMedDB again, Zhang et al. (2015) addressed interactions between cancer drugs and dietary supplements. A variation of this approach consists of using machine learning instead of the rule-based SemRep (Sang et al., 2015).

The third category of LBD systems known as *graph-based* systems are designed to enhance complex discoveries comprising multiple predicates and intermediate terms (the so-called A-n-C model). Wilkowski et al., 2011 is the first notable work exploring the A-n-C model in conjunction with graph-based methods. They use SemRep semantic predications represented as a graph, where nodes are concepts, and edges are predicates. The system suggests discoveries in the form of paths in the graph. Cameron et al., 2013; Cameron et al., 2015 employ the hierarchical agglomerative clustering (HAC) algorithm to cluster paths between two concepts (A, C). The clustering method leverages implicit semantics from MeSH descriptors and explicit semantics from the MeSH hierarchy. If semantic relatedness of paths is above a threshold, they are clustered into subgraphs that are provided as the output. Gao et al., 2019; Zhao et al., 2019 proposed two other notable graph-based LBD systems, one introducing the edge2vec model to represent graphs considering edge semantics and the other proposing an embedding-based convolutional neural network model with an attention mechanism for LBD.

With respect to the aim of evaluating an LBD system of the above cited categories, there is so far no gold standard dataset for evaluation available, and creating one is a separate challenge (Henry and McInnes, 2017). Still, several methods for validating LBD systems exist, such as replicating previous discoveries, proposing new discoveries with the following empirical evaluation (e.g., using clinical experiments), or evaluation based on time-slicing. As a matter of fact, the LBD methods and systems reviewed in this section have been applied in various studies of the biomedical domain and numerous discoveries were made through this. The following section provides an in-depth review of the respective use cases of LBD.

## 3 Literature-Based Discoveries in the Biomedical Domain

We conducted a systematic literature search for studies on LBD in the biomedical domain. We selected PubMed (2020), Google Scholar, Semantic Scholar, and ScienceDirect as the resources. We searched the resources for literature-based discovery, LBD, literature-related discovery, data-driven drug discovery, knowledge-led drug discovery, and text-based discovery (see “Search terms LBD”, Supplementary Material) for the span of 01/2020 to 05/2020. We searched the retrieved references for studies reporting cases of literature-based discoveries.

We identified 57 publications reporting one or more use cases of LBD. Each of them contains one or more discoveries, in which discovery is defined as a predicted relationship (i.e., hypothetical relationship) between two or more concepts. Due to the extensive number of discoveries cited in the publications, discoveries with identical semantic types of starting and target concepts reported in the same publication were pooled together and counted as one use case.

In all, we distinguished 91 different use cases of LBD containing one or more individual discoveries or groups of related discoveries. This sample of LBD use cases consists of pertinent or top-ranking discoveries that were explicitly highlighted in the respective publications. It’s important to note, that in several studies which identified a multitude of potential discoveries, only the cases with a high probability of occurrence, based on the criteria applied in the study, and were also specified in the corresponding publication. Moreover, many of the discoveries are re-discoveries, because in numerous studies the principal aim was the validation of their method by re-discovering confirmed relationships rather than making new discoveries. Altogether, the sample illustrates the vast diversity of literature-based discoveries reported in the biomedical domain. The selected 91 use cases are listed individually in Supplementary Table S1 (see Supplementary Material). Re-discoveries of previously published literature-based discoveries that are also mentioned in the studies were not considered. Each of the use cases was investigated for the criteria listed in Table 1. In doing so, the following notable observations can be made.

**TABLE 1:** Description of the criteria used for the investigation of the use cases of literature-based drug discovery (LBD) listed in Supplementary Table S1 (see Supplementary Material).

Criteria	Details
Discoveries	The discoveries mentioned in the LDB use cases are expressed as hypothetical relationships between two or more concepts. Discoveries with identical semantic types of starting and target concepts reported in the same publication were pooled together
Starting and target concepts	The starting concept (A term in the ABC model) and the target concept (C term) of the discoveries are elucidated and classed into semantic categories (e.g., disease, substance (incl. drug), gene, and protein) based on information in the respective study or by using the resources listed below. In many cases (e.g., closed discoveries), starting and target concepts cannot be distinguished. Therefore, this categorisation follows a conceptual perspective with the principal aim of understanding the semantic types of the concepts involved in the discoveries
Discovery pattern	The principle discovery pattern followed in the study is described, whenever possible, as ABC relationship of the related concepts (A = starting concept, B = intermediate concept, C = target concept). The concepts are classed into semantic categories based on information in the respective study or by using the resources listed below
Natural products	The presence of natural products among the concepts involved in the discoveries is investigated. Concepts are assigned to one of the following natural product categories: N=Natural product from plants, microorganisms, and higher fungi, including secondary metabolites and small molecules as well as complex mixtures such as herbal substances or extracts; ND = Natural product derivative (derivatives of the above group of natural products including semi-synthetic compounds); A = Animal venom; M = Mineral; V=Vitamin. The resources used for the classification of the concepts are listed below
Major repository	Details about the major literature resource used for establishing the knowledge database linked with the respective discovery
Underlying method	Principle approach of the method used or, if appropriate, the name of the respective LBD system
Core interest	The core interest of the LBD approach as mentioned in the respective study
LBD category	The core LBD methodology used in the respective study either as Co-occurrence, Semantic, or Graph-base

Resources used for the classification of the concepts mentioned in the discoveries: PubChem (PubChem, 2020), Medical subject headings (MESH, 2020), and ChemIDplus (ChemIDplus, 2020) of the United States National Library of Medicine; RÖMPP, online, Lexicon of pharmaceuticals and natural products (Böckler et al., 2020); Newman’s and Cragg’s 2020 review on natural products as sources for new drugs (Newman and Cragg, 2020); Patridge’s 2016 analysis of FDA, approved natural product-related drugs (Patridge et al., 2016).

Elucidating the starting and target concepts involved in the discoveries, facilitated the subsequent analysis of the underlying discovery pattern. In all 91 use cases the discovery pattern follows the ABC model in principle (A = starting concept, B = intermediate concept, C = target concept), yet the model was implemented in many different ways. There are closed discoveries with predetermined starting and target concepts, as for example the relationships between magnesium and migraine explored by Swanson, 1988 or between Alzheimer’s disease and indomethacin by Smalheiser and Swanson, 1996. Numerous other cases exhibit straightforward open discovery models, in which the starting and target concepts are specific diseases or substances, linked by heterogeneous intermediate concepts belonging to semantic types such as genes, proteins, hormones, and or biological functions. More complex models are based on multidimensional association networks or concept spaces of semantic relationships. They include examples such as the exploration of a high dimensional pharmacome-diseasome graph network (Qu et al., 2009) or the random walks in integrated networks of drug-drug similarity, disease-disease similarity, and known drug-disease associations (Chen et al., 2012).

As expected, the core interest of the LBD approach that underlies the different use cases is centred on the overarching aim of developing novel research hypotheses in the biomedical domain. The great majority of the cases aimed at predicting implicit connections between various semantic types of concepts, as for example diseases, substances, proteins, or genes. Some of the LBD approaches specifically focused on predicting either new uses for existing drugs (drug repurposing or drug reprofiling) (16 cases) or potential drug interactions (4 cases). Apart from the three studies by Swanson, 1986, Swanson, 1988, Swanson, 1990 and two earlier studies by Smalheiser and Swanson, 1994, Smalheiser and Swanson, 1996) in which the data mining was conducted manually, all studies were based on an automated LBD system.

Clearly, the most frequently used resource for establishing the knowledge database of the LBD approach used in the studies was MEDLINE or PubMed (2020) by the United States National Library of Medicine. Eighty-three of the 91 discoveries were based on information from article titles, abstracts, or full texts available in this database.

Analysing the LBD use cases for the presence of natural products among the concepts involved in the discoveries reveals that this applies to roughly one-third of the cases (30 of 91 cases). In the remaining 61 of the 91 LBD use cases, either no natural products were present among the concepts involved in the discoveries or it was not possible to establish this data, because the information available in the respective studies is unspecific 1) codified 2) or consisted of extensive lists of concepts 3) the elucidation of which is outside of the possibilities of this study (for details, see the footnotes of Supplementary Table S1, Supplementary Material). The 30 natural product-related use cases include unaltered products from plants, bacteria, or fungi (N = 17 cases), natural product derivatives (ND = 8), and animal venoms (A = 7), but also minerals (M = 6), and vitamins (V = 4). The natural products and the individual discoveries in which they are involved are specified in Supplementary Table S2 (see Supplementary Material) and discussed in the following section.

## 4 Literature-Based Discoveries Involving Natural Products

### 4.1 Investigating the Discoveries for Natural Product Specific Criteria

Twenty publications (i.e., more than one-third of the 57 publications contained in our set of literature) reported one or more discoveries that involved concepts identified as natural products in Supplementary Table S1 (see Supplementary Material). From this table, the respective discoveries were extracted and itemized. In doing so, we identified 91 individual discoveries linked with natural products. They are described in Supplementary Table S2 (see Supplementary Material). Investigating the discoveries for the criteria listed in Table 2, the following notable observation can be made.

**TABLE 2:** Description of the criteria used for the investigation of the discoveries listed in Supplementary Table S2 (see Supplementary Material).

Criteria	Details
Discoveries	Natural product-related discoveries mentioned in the respective publication are expressed as hypothetical relationships between two or more concepts
Natural product relevant concept	All natural product-related concepts mentioned in the respective discovery are listed individually
Identity of the concept	The identity of the natural product-related concept is elucidated and the concept is classified into semantic categories. Substances were classified into compound classes
Natural product category	Concepts are assigned to the same natural product categories used in Supplementary Table S1 (Supplementary Material). The categories are listed in Table 1
Natural source	The source of the natural product is indicated by using one of the following categories: plant, animal, fungus, microorganism, and mineral
Taxonomic information about the source	The taxonomic identity of the natural source is elucidated using the resources listed below. Priority is given to the source from which the natural product originally was discovered. If appropriate, additional important natural sources are listed. The taxonomic information stated in the resources was verified using Kew’s Medicinal Plant Names Services (MPNS, 2020, Medicinal Plant Names Services) or Plants of the World online (Plants of the World Online, 2020) for plants, and Global Biodiversity Information Facility (GBIF) (GBIF, 2020) for all other organisms. For plants, information about the relevant plant part (if available), as well as the family, is indicated, e.g. *Taxus brevifolia* Nutt. (bark), [Taxaceae]

In addition to the resources listed in Table 1, the following literature was used for the elucidation of the natural source: HagerROM, 2009, Hagers Enzyklopädie der Arzneistoffe und Drogen (Blaschek et al., 2009); Vardanian’s and Hruby’s Synthesis of Best-Seller Drugs (Vardanian and Hruby, 2016); Dictionary of Antibiotics and Related Substances (Bycroft and Payne, 2013).

In the 91 discoveries described, we counted 55 different natural product concepts belonging to 24 semantic classes of the biomedical or biological domain. The most important compound classes among the concepts are alkaloids (9 unique concepts), antibiotics (7), snake and other animal venoms (6), botanicals (5), and minerals (5). Assigning the 55 concepts to the five natural product categories (see Table 1) reveals the following distribution: 30 natural products from plants, microorganisms, and higher fungi, 11 natural product derivatives, 6 animal venoms, 5 minerals, and 3 vitamins.

To elucidate the natural source of these products proved to be complex in many instances. Often more than one source has to be taken into account, as illustrated by the example of mannitol. Although the sugary exudate manna from the ash species *Fraxinus ornus* L. seems to have been the original source for mannitol, the compound can also be extracted from *Laminaria cloustonii* Edmonston and other seaweed species. On the other hand, there are various additional terrestrial plants but also lichen, fungi, or insects, which produce directly or indirectly mannitol-containing types of manna. But it is unclear to what extent these sources had been exploited or whether they ever were considered for the extraction of mannitol (Harrison, 1950; Ottender and Kulling, 2019). Moreover, various compounds of plant origin are produced or co-produced by endophytic fungi (El-Elimat et al., 2014), a fact which further complicates the question about the source taxon. In any such case, we gave priority to indicate the most important source(s) from the perspective of past or present activities in research and development.

Ultimately, we were able to narrow down the source to the taxonomic level of species or genus in not less than 44 of the 50 natural products of organic source (excluding the five minerals). In the remaining six cases, the natural substances concerned are broadly distributed among higher taxonomic ranks (various families or orders) or generally in Nature. Among the source taxa of the 44 discoveries with specific taxonomic information, we counted 25 plants, 8 animals, 7 microorganisms, and 5 fungi. Since 6 of the 8 animals counted were reported in the same publication exploring the topic of animal venoms, this count must be considered with some reservation in respect of the importance of the different natural product categories. In any case, plants are the most important source of the substances mentioned in our subset of natural product-related discoveries. This finding agrees with the general interest of the natural product community in plants (Kinghorn et al., 2011).

### 4.2 Natural Products Most Frequently Involved in the Discoveries

Defining the natural products mentioned in two or more publications of the analysed literature set which hold specific taxonomic information about the source, yields the following list (ranked according to the number of discoveries involving the substance): Paclitaxel and its semi-synthetic analogue Docetaxel (10 discoveries mentioned in 5 publications), Curcumin (6 in 4), Capsaicin (5 in 2), Genistein (3 in 3), Adriamycin (Doxorubicin) (2 in 2), and Quercetin (2 in 2). The six substances, five of plant and one of microbial origin, are listed in Table 3 together with their original biological source. In Section 4.2.1, the three natural products most frequently mentioned are briefly reviewed in terms of their origin and use in medicine.

**TABLE 3:** Natural products or derivatives mentioned in two or more publications of the literature set analyzed (Data extracted from Supplementary Table S2, see Supplementary Material).

Natural product	# Disco-veries	Source taxon	References	LBD category
Paclitaxel and its analogue Docetaxel	10	*Taxus brevifolia* Nutt. and *T. baccata* L. [Taxaceae]	Baker, (2010)	Co-occurrence
			Hristovski et al., 2010	Semantic
			Ijaz et al. (2010)	Semantic
			Zhang et al. (2014a)	Semantic
			Zhang et al. (2015)	Semantic
Curcumin	6	*Curcuma longa* L. [Zingiberaceae]	Cohen et al., 2012	Semantic
			Baker, (2010)	Co-occurrence
			Zhang et al., 2014a	Semantic
			Srinivasan & Libbus, (2004)	Graph-base
Capsaicin	5	*Capsicum* spp. (mostly *C. annuum* L. and *C. frutescens* L.) [Solanaceae]	Baker, (2010)	Co-occurrence
			Wren, (2004)	Co-occurrence
Genistein	3	*Genista tinctoria* L., *Glycine max* (L.) Merr. [Fabaceae], and various other taxa	Cohen et al., 2012	Semantic
			Baker, 2010	Co-occurrence
			Ijaz et al. (2010)	Semantic
Adriamycin (Doxorubicin)	2	*Streptomyces peucetius* Grein et al	Cohen et al. (2012)	Semantic
			Zhang et al. (2014c)	Graph-base
Quercetin	2	*Quercus* ssp. [Fagaceae], and various other taxa	Cohen et al. (2012)	Semantic
			Hristovski et al. (2010)	Semantic

It is interesting to note that four of the six examples–curcumin, capsaicin, genistein, and quercetin–are compounds regarded as PAINS (Pan Assay Interference Compounds). Because these compounds tend to show non-specific activities of varying potency in a broad range of screening assays, they are usually not progressed in hit-to-lead discovery. Nevertheless, compounds with PAINS properties can have relevant activities if investigated for their target selectivity and by using the appropriate assay (Baell, 2016).

#### 4.2.1 Paclitaxel and Docetaxel

Paclitaxel (Taxol) and its semi-synthetic analogue Docetaxel currently belong to the most important anticancer drugs in clinical use. Paclitaxel, originally isolated from the bark of the Pacific yew *Taxus brevifolia* Nutt., was discovered in the 1960s as part of the NCI screening program to identify natural products that might cure cancer. The drug became approved by FDA in the 1990s for breast and ovarian cancer (Cragg, 1998). Because Paclitaxel is only contained in minor amounts in the bark of *Taxus brevifolia* Nutt. And its extraction would require the harvest of the whole tree, the commercial exploitation of the species was not feasible both from ecological and economic viewpoints. A more sustainable source for the drug was found in the needles of the European yew *Taxus baccata* L. and other yew species, which produce 10-deacetylbaccatine III. This compound can be used as a precursor for both the production of Paclitaxel as well as its semi-synthetic analogue Docetaxel (Kingston, 2011; Cragg and Pezzuto, 2016).

Paclitaxel or Docetaxel are involved in ten discoveries mentioned in five publications:

1) In Baker’s study (Baker, 2010) for predicting new uses for existing drugs based on drug-target-disease associations, the potential use of Paclitaxel in psoriasis was suggested. A subsequent cross-check of the literature by the study author confirmed the validity of the predicted relationship: Based on the previously observed improvement of psoriasis symptoms in patients on paclitaxel, a clinical trial had been conducted, and an alleviation of the symptoms was observed in all study participants;
2) Combining microarray data with semantic predications, Hristovski et al., 2005 identified Paclitaxel as one of the substances that inhibits the HSP27 gene, which is upregulated in Parkinson’s disease. The validity of the discovery is supported by the fact that this gene-disease association has already been implicated in the pathogenesis of the disease;
3) Applying a multi-level emergence model on cancer literature, Ijaz and colleagues (Ijaz et al., 2010) identified Docetaxel and Interleukin-1 as one of the high-ranking novel relationships based on the drug’s ability to increase mRNA expression of the cytokine superfamily. The lack of corresponding reports in PubMed (2020) before the publication of the study in 2010 suggests the association as being novel at the time of its discovery;
4) In the drug-gene-cancer discovery pathway applied by Zhang et al., 2014c paclitaxel was one of the substances predicted as a potential candidate in the treatment of prostate cancer. While an association between Paclitaxel and prostate cancer is known from previous experimental studies, the discovery revealed the drug’s ability to upregulate the expression of the Fas receptor that contributes to the induction of apoptosis;
5) Another study by Zhang et al., 2015 based on the same discovery approach, aimed at identifying interactions between cancer drugs and dietary supplements, especially botanicals. Focusing on examples exhibiting an influence on the Cytochrome P450 gene family, 14 potential interactions of this kind are revealed in the paper. Seven of these were previously unknown associations: Herbal preparations of Echinacea and the cancer drugs Toremifine or Exemestane; Grape seed extracts and Docetaxel; Herbal preparations of Ginseng and Docetaxel; Vitamin E and Prednisone or Cyclophosphamide; Melatonin and Docetaxel.

#### 4.2.2 Curcumin

Curcumin, a mixture of curcuminoids, is a major secondary metabolite in the bright yellow spice turmeric obtained from the rhizome of *Curcuma longa* L., a Zingiberaceae species endemic to the Indian subcontinent. Turmeric is not only an important ingredient in Asian or particularly Indian cuisine but also holds a prominent position in local traditional medicines. It is used in the treatment of wounds, ulcers, infections, jaundice, urinary tract diseases, and various other conditions. Turmeric’s anti-inflammatory profile previously observed in clinical applications has gained experimental support by the demonstrated inhibitory effects of curcumin against the signaling cascade of activated NF-κB (Bremner and Heinrich, 2005). Preclinical data point to curcumin’s potential in cancer, cardiovascular, inflammatory, metabolic, neurological, and skin diseases as well as its ability to modulate the immune system. However, many limitations have been recognized for the further development of the compound and its oral bioavailability observed in clinical studies is largely insufficient (Nelson et al., 2017; Catanzaro et al., 2018). In addition, the PAINS properties of curcumin challenge the significance of its activities observed and consequently also the respective discoveries made by LBD systems. A more systemic approach taking into account the chemical and pharmacological complexity of multi-component mixtures (Nelson et al., 2017) or their influence on the human microbiome might be revealing here.

Curcumin is involved in six discoveries mentioned in four studies:

1) Baker’s (2010) re-profiling approach (see above). In this study, curcumin was suggested as a potential corrector of the protein misfolding often observed in the development of cystic fibrosis. However, the validity of this discovery was considered uncertain as clinical phase I trials had so far been negative;
2) In Cohen’s et al. (2012) predication-based semantic indexing approach curcumin ranked under the top 20 predictions of compounds with a therapeutic potential in multiple myeloma. Evidence supporting this hypothesis was found in a corresponding *in vitro* assay with curcumin analogues;
3) Using an open discovery algorithm, Srinivasan and Libbus (2004) found previously unknown associations between curcumin and retinal diseases (including diabetic retinopathy, inflammation, and glaucoma), Crohn’s disease, as well as disorders related to the spinal cord. The predicted associations are based on indirect connections involving TNF-α, MAPK, NF-κB, COX-2, and other cytokines or interleukins. A cross-check of the literature by the study authors was found to provide genetic and biochemical evidence for a potential benefit of curcumin in the treatment of these conditions;
4) In the above-mentioned study by Zhang et al., 2014a curcumin was also one of the 18 substances predicted as potential novel candidates in the treatment of prostate cancer. The underlying mechanisms include curcumin’s ability to promote levels of the Fas receptor and the signal-transducing adaptor protein FADD, both well known for their role in apoptosis.

#### 4.2.3 Capsaicin

Capsaicin, an alkylamide, is the pungent constituent contained in chili, the fruits of *Capsicum* species most importantly *C. annuum* L. or *C. frutescens* L. Chili has a millennia-old traditional use in Meso- and Southern American cultures as a spice but also as a medicine, for example in inflammatory conditions (Heinrich, 2010). The discovery of capsaicin’s role as a ligand for one of the sensory receptors responsible for pain transmission turned this culinary compound into a crucial mediator for the study of neurogenic inflammation and pain (Calixto et al., 2005). Today, capsaicin is used as a topical analgesic especially for pain derived from neuropathic conditions. Injectable purified forms of the compound are also under clinical evaluation (Calixto et al., 2005; Frias and Merighi, 2016).

Capsaicin is involved in five discoveries mentioned in two studies:

1) Two of the discoveries were reported in Baker’s (2010) re-profiling approach above-mentioned. Here, the substance was predicted as a potential therapy not only for migraine but also for psoriasis. The validity of both discoveries was confirmed by the analysis of the corresponding literature: *i*) Based on capsaicin’s ability to activate the vanilloid receptors resulting in desensitization of the nerve fibers, the compound was tested in a small clinical trial with migraine patients and revealed generally positive results. *ii*) Capsaicin’s efficacy in reducing the itch associated with psoriasis was demonstrated in a double-blind controlled study;
2) The other three discoveries involving capsaicin were all mentioned in Wren’s (2004) open discovery approach based on a mutual information measure model. First, an association between capsaicin and the ileum was predicted. This link is based on the fact that the ileum is frequently used as a model to test capsaicin’s effect on muscle contraction. Further, the study also predicted associations between capsaicin and the alkaloid atropine, found in *Atropa belladonna* L. and other Solanaceae, as well as between capsaicin and tetrodotoxin, a highly toxic compound isolated from the Japanese pufferfish *Takifugu rubripes* (Temminck and Schlegel, 1850). Atropine and tetrodotoxin are antagonists blocking the afferent nerve transmission in response to capsaicin.

Our sample of the natural products most frequently involved in the discoveries also includes the antibiotic Adriamycin (Doxorubicin), the isoflavone genistein, and the flavonol quercetin. The discoveries involving Adriamycin point to the compound’s possible potential in multiple myeloma (Cohen et al., 2012) or its association with the sympathomimetic drug Dobutamine used in cardiac conditions (Zhang Y. et al., 2014). The latter is supported by the observation that chemotherapy side effects, for example of Adriamycin, may increase the risk of heart disease in cancer patients, which in turn might be prevented by Dobutamine. For genistein, a potential therapeutic use in cystic fibrosis (Baker, 2010) or multiple myeloma (Cohen et al., 2012), as well as its ability to increase apoptosis in the HCT-116 human colon carcinoma cell line (Ijaz et al., 2010) was predicted. Quercetin was likewise identified as one of the substances influencing the development of multiple myeloma (Cohen et al., 2012), but also genes associated with Parkinson’s disease (Hristovski et al., 2010).

## 5 Opportunities and Challenges of LBD With Natural Products

### 5.1 Re-discoveries and Novel Discoveries

As illustrated by our sample of 91 use cases (Supplementary Table S1, see Supplementary Material) there is a broad range of possible applications of LBD systems and a vast diversity of predicted discoveries in the biomedical domain. In the 91 natural product-related discoveries (Supplementary Table S2, see Supplementary Material), we identified 55 different natural products ranging from plants, fungi, and microorganisms or their compounds to animal venoms, minerals, and vitamins. As shown by the examples of Paclitaxel/Docetaxel, curcumin, capsaicin, genistein, Adriamycin (Doxorubicin), and quercetin there is a real potential for LBD with natural products. The review of the discoveries involving these most frequently cited natural products illustrates that the LBD systems are enabled to identify true associations between concepts by re-discovering established clinical facts. In fact, rather than finding novel associations, the principal aim of the discovery approaches in our natural product-related data set (Supplementary Table S2, see Supplementary Material) was to validate the LBD systems used by retrieving established knowledge and thereby substantiate the potential of LBD in this context. Nevertheless, our data set also included a few discoveries that have not been reported in the literature prior to the date of the respective publication. These examples point to the potential of finding novel drug leads or formulating new hypotheses. In the list of the most frequently cited natural products (Table 3), we identified three corresponding cases: 1) Associations between curcumin and retinal diseases, Crohn’s disease or disorders of the spinal cord (Srinivasan and Libbus, 2004); 2) Interactions between cancer drugs (Toremifine, Exemestane, Docetaxel, Prednisone or Cyclophosphamide) and dietary supplements (Vitamin E, Melatonin) or botanicals (preparations of Echinacea, Grape seed, and Ginseng (Zhang et al., 2015); 3) The relationship between Interleukin-1 and Docetaxel by the former’s ability to increase mRNA expression of the cytokine superfamily (Ijaz et al., 2010).

### 5.2 High Potential Topics for LBD With Natural Products

Based on the results of this review, we consider in particular the following topics as potentially rewarding areas of application for LBD with natural products: Drug discovery (including therapeutic or toxicological profiling), drug repurposing, mode of action as well as undesired or synergistic interactions between drugs and/or substances. To illustrate our argument, we here provide corresponding examples selected from the discoveries analysed or suggest conceptual ideas about potential applications in these areas.

#### 5.2.1 Drug discovery

Using an open discovery approach Srinivasan and Libbus (2004) aimed at finding unknown potential uses for curcumin (starting concept) based on information on the compound’s influence on genes or gene products (intermediate concept) associated with specific diseases (target concept). Several corresponding uses were identified and the fact that none of them was reported in the public literature prior to the date of the study, points to the novelty of the discoveries. Various other discovery pathways with the same types of starting and target concepts could be developed, as for example a molecular target-based approach, in which the intermediate-term can include receptors or ligands, or a physiological approach, in which the intermediate-term is a biological process.

#### 5.2.2 Drug repurposing

Li et al., 2009 developed a model of a disease-specific molecular connectivity map with the aim to find indirect connections between Alzheimer’s Disease (starting concept) and drugs from non-related therapeutic areas (target concept) based on associated molecular data (intermediate concept). They found that quinidine, a stereoisomer of quinine contained in the bark of *Cinchona officinalis* and other infrageneric taxa (Böckler et al., 2020), might be a potential drug candidate in context with dementia. As argued in the study, the predicted relationship was based on quinidine’s use as an antiarrhythmic agent and epidemiological data pointing to a potential role of vascular risk factors and the development of Alzheimer’s Disease.

#### 5.2.3 Mode of action

In some instances, effective drug therapies are applied in clinical practice without having a clear understanding of their mode of action. Taking the example of the use of antipsychotic drugs in the treatment of cancer, Ahlers et al., 2007 illustrate how an LBD approach based on semantic predications can facilitate the elucidation of the mechanisms underlying drug therapies.

#### 5.2.4 Interactions

The risk of drug-supplement interactions between cancer drugs and dietary supplements, especially botanicals, was explored by Zhang et al. (2015). By applying enhanced machine-learning-based filtering on their LBD system built on semantic predications, the researchers found known interactions but also inferred several unknown potential interactions.

Similar to exploring undesirable interactions, the search for synergistic interactions between drugs and natural products could be pursued by a correspondingly tailored LBD system. For example, the application of an open discovery approach with phytochemicals (starting concept) could predict that certain phytochemicals interacted with specific targets (first intermediate concept), which are related to a particular disease (second intermediate concept) that is treated with a specific drug (target concept). The combination of the concerned phytochemical(s) with the particular drug might improve drug therapy through a synergistic multi-target effect. Drug combinations play an important role especially in the therapy of complex diseases, such as in highly active antiretroviral therapy (HAART) (Pereira and Paridaen, 2004) or cancer therapy (Peters et al., 2000). Various potentiating interactions involving natural products, where the combination of two or more compounds resulted in additive or synergistic effects, are reported in the literature (Schmidt et al., 2008; Li and Vederas, 2009).

### 5.3 Pre-conditions and Challenges for the Application of LBD

Of course, natural product research using LBD systems will not escape the typical challenges linked with drug discovery from Nature, such as, in the first place, securing a sustainable solution for the resupply of the natural product (Atanasov et al., 2015; Newman and Cragg, 2016). In addition to this, there are circumstances specific to this kind of data mining discovery system. First of all, the pieces of data or knowledge, which inform the starting, intermediate, and target concepts of a discovery pathway must be published and accessible. This is the basic precondition for the realization of any LBD approach. Further, the fact of dealing with published information involves the risk of the LBD system producing known associations between the concepts investigated; hence, of discovering what has already been described and possibly put under patent protection. However, while there is a high risk of duplication also in the classical drug discovery process based on bioassay-guided lead identification (Li and Vederas, 2009), in an established LBD system the effort wasted in such a case is comparatively low and does not involve costly laboratory experiments. Moreover, drug discovery with automated LBD commonly involves a dereplication step consisting of separating out the previously known associations. Excluding known substances early in the drug discovery process is of equal importance in laboratory-based natural product screening programs, as illustrated by the various existing dereplication strategies (Hubert et al., 2017).

Other points to be considered include the precision of the automated LBD system used, hence the proportion of relevant documents or semantic predications from the results retrieved and the effectiveness of possibly applied filters. In this respect, the issue of PAINS, as mentioned above, provides an incentive to equip LBD systems with possibilities to selectively identify and separate corresponding compounds. Developing appropriate filters to detect PAINS and other compounds giving rise to artefacts, but also to discern highly promiscuous compounds with interesting pharmacological activities, are of critical importance for small molecule drug development (Gilberg et al., 2016; Baell and Nissink, 2018). Not to be underestimated is the complexity of the subsequent manual review of presumably relevant associations produced by an LBD system and their interpretation by domain experts.

Finally, literature-based discoveries are predictions based on hypothetical relationships between the involved concepts that first need to be verified by further investigations. This can pose a significant challenge both in terms of establishing an experimental model for furnishing proof as well as in terms of investment. However, these expenditures should be considered in relationship with the advantages offered by an LBD system in supporting the discovery process by recontextualizing pre-existing information. The knowledge generated based on this can contribute substantially to various work steps and challenges on the path from lead to candidate drug, irrespective of whether these are linked to pharmacology and toxicology or pharmacokinetics and pharmacodynamics.

## 6 Conclusions

Today, researchers have access to an immense collection of rapidly expanding biological, molecular, pharmacological, or medicinal information. A countless number of natural product-specific databases of diverse thematic scopes ranging from pre-selected pools of specific classes of natural products to chemical, pharmacological and ethnobotanical data on vascular plants exist. However, most of the data is maintained in detached data warehouses, and this hampers any contextualization required for better exploiting this wealth of knowledge.

Literature-based discovery (LBD) is a research field aiming at finding unknown connections between concepts by analysing literature. The discovery process can be performed manually through literature screening, as it is still done in many domains; however, the process is laborious and time-consuming. Various systems of automated LBD exist today, which analyse connections between terms based on their co-occurrences, semantic relations expressed in texts, or on vector representations of texts. Therefore, automated LBD lends itself as an efficient tool of high potential for contextualizing detached pieces of information to formulate new hypotheses for research and development. Moreover, the insights gained through the evaluation of the implicit connections established by automated LBD between previously non-associated pieces of information imply that this kind of system can facilitate the often lengthy and expensive process of lead identification, optimization, and development as well as contribute to various other aspects in drug development. Using an appropriate representation of data, natural language text can also be integrated and queried with structured data stored in databases relevant in this context (Sima et al., 2019a; Sima et al., 2019b; Koroleva et al., 2020).

Although automated LBD has so far not been introduced to the domain of natural products, the examples in our data set illustrate the potential of this hitherto little tapped approach. We argue that automated LBD holds promising opportunities for the natural product research community, especially in context with drug discovery or drug repurposing, the elucidation of the mode of action, or the exploration of undesired or synergistic interactions. However, it still needs to be evaluated systematically to what extent automated LBD can support natural product drug discovery.
