# Supplementary material for: Computational Literature-based Discovery for Natural Products Research: Current State and Future Prospects
Source: Front Bioinform. 2022 Mar 15;2:827207. doi: 10.3389/fbinf.2022.827207 (PMC9580913; doi:10.3389/fbinf.2022.827207)
Supplement: Supplementary file 1 [file DataSheet2.PDF]

Supplementary Material – Supplementary Tables 1 and 2

Article title: Computational literature-based discovery for natural products research: current state and future prospects

Authors: Andreas Lardos, Ahmad Aghaebrahimian, Anna Koroleva, Julia Sidorova, Evelyn Wolfram, Maria Anisimova, Manuel Gil

Supplementary Table 1. Overview of use cases of literature-based discoveries reported in the biomedical domain (The table is sorted, first, according to "LBD category" and, secondly, according to "Starting and target concept").

Discoveries	LBD Category	Starting and target concepts	Discovery pattern	Natural products	Major repository	Underlying method	Core interest	Ref.
Relationships between enzymes, substrates and signal proteins in the calpain signal transduction pathway	Co-occurrence	Biological process / Biological process	ABC relationships based on user-defined dictionaries of bio-medical concepts	Not applicable	MEDLINE titles and abstracts	TansMiner system (principle of transitive closure applied on the association graph)	Novel research hypothesis in biomedicine	28
Associations between dephostatin or damnacanthal and cell proliferation	Co-occurrence	Biological process / Substance	Disease→Gene→ Disease	N	MEDLINE titles and abstracts, MeSH terms	BITOLA (interactive biomedical discovery support system)	Candidate genes for the interaction between diseases	15
Association between deafness and macular degeneration based on their co-occurrence in literature on muscular dystrophy	Co-occurrence	Disease / Disease	Exploration of the neighbourhood of a concept in the concept space	Not applicable	MEDLINE abstracts	Multidimensional associative concept space (ACS)	Novel relationships between biomedical concepts	47
Association between Alzheimer's disease and endocannabinoids	Co-occurrence	Disease / Endogenous molecule	ABC relationships with ranking of the target terms by the number of linking terms that con-nect to the starting term	Not applicable	MeSH terms of MEDLINE citations	LBD system LitLinker incorporating knowledge-based methodologies with statistics	Novel connections between diseases and drugs, chemicals, genes or molecular sequences	53

Discoveries	LBD Category	Starting and target concepts	Discovery pattern	Natural products	Major repository	Underlying method	Core interest	Ref.
Genes associated with Grave's disease	Co-occurrence	Disease / Gene	ABC relationships of diseases, sub-stances, genes, pathways and biol. processes	Not applicable	MEDLINE abstracts	CoPub Discovery	Novel associations between genes, diseases, drugs and biological processes	15
Association between Alzheimer's disease and estrogen	Co-occurrence	Disease / Hormone	ABC relationship of Alzheimer's disease & estrogen	Not applicable	MEDLINE titles	ARROWSMITH two node search	Novel links between estrogen and Alzheimer's disease	37
Association between schizophrenia and secretin	Co-occurrence	Disease / Hormone	ABC relationships with ranking of the target terms by the number of linking terms that connect to the starting term	Not applicable	MeSH terms ofMEDLINE citations	LBD system LitLinker incorporating knowledge-based methodologies with statistics	Novel connections between diseases and drugs, chemicals, genes or molecular sequences	53
Association between type 2 diabetes and epigenetic factors	Co-occurrence	Disease / Phenotype	ABC relationships with ranking of the relationships against a random network model	Not applicable	MEDLINE titles and abstracts	IRIDESCENT (implicit relationship identification by in silico construction of an entity-based network from text)	Novel factors implicated in the pathology of type 2 diabetes	52
Association between schizophrenia and calcium independent phospholipase A2 based on Vitamin E	Co-occurrence	Disease / Protein	ABC relationship of schizophrenia and calcium independent phospholipase A2	V	MEDLINE titles	ARROWSMITH two node search	Novel links between schizophrenia and calcium independent phospholipase A	38
Association between DDX3 (dead box RNA helicase 3) and cancer adhesion or metastasis	Co-occurrence	Disease / Protein	Similarity network of biomedical papers	Not applicable	MEDLINE citations	MOLIERE (weighted multi-layered network, hypothesis generation system)	Drug12 repurposing	44

**Discoveries**	**LBD Category**	**Starting and target concepts**	**Discovery pattern**	**Natural products**	**Major repository**	**Underlying method**	**Core interest**	**Ref.**
Association between migraine and AMPA receptors	Co-occurrence	Disease / Receptor	ABC relationships with ranking of the target terms by the number of linking terms that connect to the starting term	Not applicable	MeSH terms of MEDLINE citations	LBD system LitLinker incorporating knowledge-based methodologies with statistics	Novel connections between diseases and drugs, chemicals, genes or molecular sequences	53
Drugs, natural products and minerals with a potential in cystic fibrosis, migraine or psoriasis	Co-occurrence	Disease / Substance	Disease→Protein→ Substance	N, ND, M	MEDLINE citations annotated with MeSH terms	ChemoText database on drug-target-disease associations	Prediction of new uses for existing drugs (Drug reprofiling)	3
Association between Alzheimer's disease and Indomethacin	Co-occurrence	Disease / Substance	ABC relationship of Alzheimer's disease and indomethacin	Not applicable	MEDLINE titles	Systematic manual search	Novel links between Indomethacin and Alzheimer's disease	36
Association between Raynaud's disease and fish oil	Co-occurrence	Disease / Substance	ABC relationship of Raynaud's disease and fish oil	N	MEDLINE and Embase titles	Manual search and synthesis of related but not interacting literatures	Identification of potentially useful and possibly new hypothesis	41
Association between migraine and magnesium	Co-occurrence	Disease / Substance	ABC relationship of migraine and magnesium	M	MEDLINE titles	Manual search and synthesis of related but not interacting literatures	Identification of potentially useful and possibly new hypothesis	42
Association between cardiac hypertrophy and chlorpromazine	Co-occurrence	Disease / Substance	ABC relationships with ranking of the relationships against a random network model	Not applicable	MEDLINE titles and abstracts	Analysis of shared relationships of concepts scored against a random network model	Undiscovered relationships between different biomedical concepts	50

Discoveries	LBD Category	Starting and target concepts	Discovery pattern	Natural products	Major repository	Underlying method	Core interest	Ref.
Relationships between genes associated with breast cancer	Co-occurrence	Gene / Gene	ABC relationships based on user-defined dictionaries of bio-medical concepts	Not applicable	MEDLINE titles and abstracts	TansMiner system (principle of transitive closure applied on the association graph)	Novel research hypothesis in biomedicine	28
Association between insulin and ferritin based on their cooccurrence in literature on muscular dystrophy	Co-occurrence	Hormone / Protein	Exploration of the neighbourhood of a concept in the concept space	Not applicable	MEDLINE abstracts	Multidimensional associative concept space (ACS)	Novel relationships between biomedical concepts	47
Association between somatomedin C and arginine	Co-occurrence	Protein / Substance	ABC relationship of somatomedin C and arginine	Not applicable	MEDLINE titles	Manual search and synthesis of related but not interacting literatures	Identification of neglected potentially fruitful areas of research	43
Association between convulxin and the antiaggregatory effect, or the aggregability of blood platelets	Co-occurrence	Substance / Biological process	Network of Semantic relationships	A	MEDLINE abstracts	Reflective Random Indexing (RRI)	Discovery of implicit connections in the biomedical domain	11
Additional biological processes associated with Pitavastatin	Co-occurrence	Substance / Biological process	ABC relationships of diseases, Substances, genes, pathways and biological processes	Not applicable	MEDLINE abstracts	CoPub Discovery	Novel associations between genes, diseases, drugs and biological processes	15
Milnacipran with a potential in various disorders, syndromes and symptoms	Co-occurrence	Substance / Disease	ABC relationships of diseases, Substances, genes, pathways and biological processes	Not applicable	MEDLINE abstracts	CoPub Discovery	Novel associations between genes, diseases, drugs and biological processes	15
Association between magnesium deficiency and	Co-occurrence	Substance / Disease	ABC relationship of magnesium and	M	MEDLINE titles and	Systematic manual search	Influence of magnesium levels on	35

Discoveries	LBD Category	Starting and target concepts	Discovery pattern	Natural products	Major repository	Underlying method	Core interest	Ref.
neurologic disease			nervous system diseases		abstracts, MeSH terms		brain function or neurologic diseases	
Association between convulxin and citrated compounds	Co-occurrence	Substance / Endogen. molecule	Network of semantic relationships	A	MEDLINE abstracts	Reflective Random Indexing (RRI)	Discovery of implicit connections in the biomedical domain	11
Association between Venlafaxine and the genes HTR1A and HTR2A	Co-occurrence	Substance / Gene	Similarity network of biomedical papers	Not applicable	MEDLINE citations	MOLIERE (weighted multi-layered network)	Discover implicit connections between drug and gene	44
Animal taxa containing in their venom cobratoxin or related toxins; Animal taxa containing in their venom convulxin or related toxins	Co-occurrence	Substance / Organism	Network of semantic relationships	A	MEDLINE abstracts	Reflective Random Indexing (RRI)	Discovery of implicit connections in the biomedical domain	11
Association between convulxin and thrombospondin or the glycoproteins ib, iib, iiia or v	Co-occurrence	Substance / Protein	Network of Semantic relationships	A	MEDLINE abstracts	Reflective Random Indexing (RRI)	Discovery of implicit connections in the biomedical domain	11
Association between arsenic and autotaxin via Nrf2 and other genes	Co-occurrence	Substance / Protein	ABC relationships applied on the closed discovery of arsenic and autotaxin	M	MEDLINE titles and abstracts	LION LBD (literature based discovery system for cancer biology)	Identification of undiscovered links in the biomedical literature	31
Drugs predicted to be 5-HT6 receptor binders or dopamine antagonists based on their side effects profile	Co-occurrence	Substance / Receptor	Substance→Molecular activity→Receptor	Not applicable	NIMH Psychoactive drug screen. Program, Ki, MeSH	ChemoText database on drug-target-disease associations (filtered by data on side effects)	Prediction of drug molecular activity from side effects (Drug reprofiling)	3

**Discoveries**	**LBD Category**	**Starting and target concepts**	**Discovery pattern**	**Natural products**	**Major repository**	**Underlying method**	**Core interest**	**Ref.**
Association between cobratoxin and the acetylcholine receptor or the nicotinic acetylcholin-esterase receptor	Co-occurrence	Substance / Receptor	Network of semantic relationships	A	MEDLINE abstracts	Reflective Random Indexing (RRI)	Discovery of implicit connections in the biomedical domain	11
Association between cobratoxin and the neurotoxin erythroidine, other animal venoms, suberyl-dicholine, or the maleimid scafold; Association between con-vulxin and the anticoagulants hirudin and apyrase or ristocetin	Co-occurrence	Substance / Substance	Network of Semantic relationships	A / N	MEDLINE abstracts	Reflective Random Indexing (RRI)	Discovery of implicit connections in the biomedical domain	11
Association between capsaicin and various drugs	Co-occurrence	Substance / Substance	ABC relationships with ranking asso-ciations based on MIM	N	MEDLINE titles and abstracts	Mutual information measure (MIM) model of shared associations	Extension of the calculation of mutual information to indirect associations of biomedical concepts	51
Association between cobratoxin and electric fish muscle fibres; Association between convulxin and platelet-rich plasma	Co-occurrence	Substance / Tissue	Network of Semantic relationships	A	MEDLINE abstracts	Reflective Random Indexing (RRI)	Discovery of implicit connections in the biomedical domain	11
Ileum used in experiments to test capsaicin's effect on muscle contraction	Co-occurrence	Substance / Tissue	ABC relationships with ranking associations based on MIM	N	MEDLINE titles and abstracts	Mutual information measure (MIM) model of shared associations	Information to in-direct associations of biomedical concepts	51

Discoveries	LBD Category	Starting and target concepts	Discovery pattern	Natural products	Major repository	Underlying method	Core interest	Ref.
Genes involved in long term potentiation	Graph-base	Biological process / Gene	Gene-protein-Substance network	Not applicable	MEDLINE titles and abstracts	Chilibot (natural language processing-based text mining)	Generation of new hypothesis in biological research	7
Association between hair cycle and fibroblast growth factor 5	Graph-base	Biological process / Protein	ABC relationships of different biomedical concepts	Not applicable	MEDLINE citations	EpiphaNet (interactive visual representation of concept associations)	Identification and exploration of associations of biomedical concepts	10
Association between various proteins and senescence	Graph-base	Biological process / Protein	ABC relationships of proteins and diseases	Not applicable	MEDLINE titles and abstracts	LION (LBD system for cancer biology)	Undiscovered links in the molecular biology of diseases	13
Association between Down syndrome and cell polarity	Graph-base	Disease / Biological process	ABC relationship of Down syndrome and cell polarity with relational frequent patterns	Not applicable	MEDLINE abstracts	Inductive Logic Programming (ILP) technique using the algorithm WARMR in the data mining system ACE	Discovery of meaningful knowledge in addition to the indirect connections	45
Genes associated with prostate cancer and tumor growth; Genes associated with communicable diseases, viral infections or Castelman's disease	Graph-base	Disease / Disease	Integrated Substance-disease-gene network	Not applicable	MEDLINE titles and abstracts	Semantic predications (SemRep, Semantic MEDLINE), network topology analysis	Investigation of disease-drug-gene associations; Case study 1) Prioritization of disease genes	56
Genes associated with kidney diseases and skin disorders	Graph-base	Disease / Disease	Integrated Substance-disease-gene network	Not applicable	MEDLINE titles and abstracts	Semantic predications (SemRep, Semantic MEDLINE), network topology analysis	Case study 2) Disease relationship inference	56

Discoveries	LBD Category	Starting and target concepts	Discovery pattern	Natural products	Major repository	Underlying method	Core interest	Ref.
Association between diminished sleep quality in aging men, testosterone and cortisol	Graph-base	Disease / Hormone	ABC relationships of hormones and disorders	Not applicable	MEDLINE titles and abstracts	Semantic predications (SemRep, Semantic MEDLINE)	Novel research hypothesis in biomedicine	27
Association between adenoma, pancreatic or thyroid cancer and various proteins	Graph-base	Disease / Protein	ABC relationships of proteins and diseases	Not applicable	MEDLINE titles and abstracts	LION (LBD system for cancer biology)	Undiscovered links in the molecular biology of diseases	13
Association between sarco-idosis and various peptides	Graph-base	Disease / Protein	Disease→B→Protein	Not applicable	MEDLINE abstracts	Knowledge graph network analysis	Drug repurposing	16
Association between prostate carcinoma and vitamin D receptor through prostate specific antigen	Graph-base	Disease / Receptor	ABC relationships of different biomedical concepts	Not applicable	MEDLINE citations	EpiphaNet (interactive visual representation of concept associations)	Identification and exploration of associations of biomedical concepts	10
Imatinib with a potential in Creutzfeldt–Jakob disease or pulmonary arterial hypertension	Graph-base	Disease / Substance	Disease→B→Substanc e	Not applicable	MEDLINE abstracts	Knowledge graph network analysis	Drug repurposing	16
Diltiazem and Quinidine and their potential in Alzheimer's disease	Graph-base	Disease / Substance	Disease specific substance-protein connectivity maps	N	MEDLINE abstracts	Molecular connectivity map development approach	Drug repurposing	25
Drugs with a potential in Alzheimer's disease	Graph-base	Disease / Substance	Integrated network of substance-substance similarity, disease-disease similarity, and known substance-disease association	N, ND	SMILES, OMIM database, DrugBank	Two-pass random walk with restart on the drug-disease heterogeneous network	Drug repositioning	26

Discoveries	LBD Category	Starting and target concepts	Discovery pattern	Natural products	Major repository	Underlying method	Core interest	Ref.
Tamoxifen with a potential in systemic Lupus erythematosus	Graph-base	Disease / Substance	Integrated network of disease, Substances, genes, pathways and clinical features	Not applicable	Multiple public drug or gene databases	High-dimensional drug-disease graph network based on the designed knowledge framework DDCO (Disease Drug Correlation Ontology)	Drug repurposing	32
Association between drugs used in nervous system disorders with Alzheimer's disease	Graph-base	Disease / Substance	Integrated Substance disease- gene network	Not defined[e]	MEDLINE titles and abstracts	Semantic predications (SemRep, Semantic MEDLINE), network topology analysis	Case study 3) Drug repositioning	56
IFN-γ and vaccine-mediated gene interactions	Graph-base	Gene / Gene	Gene-gene network	Not applicable	MEDLINE abstracts	Natural language processing and network centrality analysis	Discovery of gene interactions and hypotheses generation	29
Twenty-five gene pairs and associated diseases	Graph-base	Gene / Gene	Gene-citation-gene network	Not applicable	MEDLINE abstracts	Entity metrics model applied on constructed gene-citation-gene network	Detection of implicit gene interactions	39
Association between clock genes and norepinephrine through melatonin and the cyto-kines IL-1 and IL-6 in context with depressive disorders	Graph-base	Gene / Hormone	ABC relationships considering the B term as a subchain of inter-mediate concepts	Not applicable	MEDLINE citations	Semantic predications (provided by SemRep system) and graph based methods	Exploration of poorly understood relationships in biomedicine	49
Association between s100a9and cyclin-dependent kinaseinhibitor 2a	Graph-base	Protein / Protein	ABC relationships of different biomedical concepts	Not applicable	MEDLINE citations	EpiphaNet (interactive visual representation of concept associations)	Identification and exploration of associations of biomedical concepts	10

Discoveries	LBD Category	Starting and target concepts	Discovery pattern	Natural products	Major repository	Underlying method	Core interest	Ref.
Association between various proteins	Graph-base	Protein / Protein	ABC relationships of proteins and diseases	Not applicable	MEDLINE titles and abstracts	LION (LBD system for cancer biology)	Undiscovered links in the molecular biology of diseases	13
Association between calgranulin a and sin-1a	Graph-base	Protein / Substance	ABC relationships of different biomedical concepts	Not applicable	MEDLINE citations	EpiphaNet (interactive visual representation of concept associations)	Identification and exploration of associations of biomedical concepts	10
Imatinib as a potential treatment in asthma	Graph-base	Substance / Disease	Substance→Target→ Disease	Not applicable	MEDLINE citations annotated by MeSH terms	ChemoText web server based on drug-target-disease associations	Drug repurposing and annotating clinical outcome pathways	6
Associations between Chlor-promazine and psychosis, Celecoxib and colorectal cancer, Mifepristone and psychotic major depression	Graph-base	Substance / Disease	Substance-target-protein-disease network	Not applicable	Multiple public drug or gene databases	Ondex data integration workflow	Drug repurposing	9
Association between curcumin and retinal diseases, Crohn's disease or disorders related to the spinal cord	Graph-base	Substance / Disease	ABC relationships of the starting concept curcumin	N	MEDLINE citations, MeSH terms	Open discovery process built on the notion of topics and their profiles	Discovery of novel uses for curcumin	40
Several drug-target interactions	Graph-base	Substance / Protein	Integrated network of protein-protein similarity, Substance-Substance similarity and known Substance-target interaction	Not defined	Data downloaded from Yamanishi 2008	Network-based random walk with restart on the heterogeneous network	Prediction of potential drug target interactions	8

Discoveries	LBD Category	Starting and target concepts	Discovery pattern	Natural products	Major repository	Underlying method	Core interest	Ref.
Association between chemotherapy side effects of Doxorubicin and Dobutamine used in heart diseases	Graph-base	Substance / Substance	Substance→Gene→ Substance or Substance→Gene→ Biological function←Gene← Substance	N	MEDLINE titles and abstracts	Semantic predications (SemRep, Semantic MEDLINE)	Drug-drug interactions	56
Association between action of nitric oxide on mitochondria and function of post-synaptic dens.	Semantic	Biological process / Biological process	ABC relationships with ranking of the B terms according to their likely relevance	Not applicable	MEDLINE citations	ARROWSMITH two node search including a logistic regression model for estimating the probability of relevance for B terms	Identification of implicit links in two disparate sets of articles	46
Targets in the Vitamin D meta-bolism pathway to aid novel Vitamin D analogue design	Semantic	Biological process / Hormone	Substance→Gene→ Disease	Not applicable	MEDLINE citations, SemMedDB	Semantic predication	Drug repositioning	34
Interaction between Ginseng and Ginko biloba extract	Semantic	Botanical / Botanical	Botanical→Gene→ Botanical	N	MEDLINE titles and abstracts	Semantic predications (SemRep, Semantic MEDLINE)	Interactions between cancer drugs and dietary supplements	57
Interactions between Echinacea, Grape seed extract Kava preparation or Ginseng and cancer drugs	Semantic	Botanical / Substance	Botanical→Gene→ Substance	N, ND	MEDLINE titles and abstracts	Semantic predications (SemRep, Semantic MEDLINE)	Interactions between cancer drugs and dietary supplements	57
Association between retinal detachment and aortic aneurysm	Semantic	Disease / Disease	ABC relationships with ranking of the B terms according to their likely relevance	Not applicable	MEDLINE citations	ARROWSMITH two node search including a logistic regression model	Identification of implicit links in two disparate sets of articles	46

Discoveries	LBD Category	Starting and target concepts	Discovery pattern	Natural products	Major repository	Underlying method	Core interest	Ref.
Association between amyotrophic lateral sclerosis and gene mutations	Semantic	Disease / Gene	ABC relationships of cells, Substances, diseases and organisms	Not applicable	MEDLINE titles and abstracts	Context-based and context-assignment-based models	Generation of new hypotheses of relations between cells, drugs, diseases and organisms	22
Genes associated with bilateral perisylvian polymicrogyria disease	Semantic	Disease / Gene	Disease→Cell function →Gene	Not applicable	MEDLINE titles and abstracts, MeSH terms	BITOLA (interactive biomedical discovery support system)	New relations between diseases and genes	17, 18
Association between Huntington's disease and insulin	Semantic	Disease / Hormone	Disease→Hormone or Biological function→ Substance	Not applicable	MEDLINE titles and abstracts, MeSH terms	Semantic predications, LBD system BITOLA	Uncovering new associations	19
Association between Alzheimer's disease and protein-protein interactions	Semantic	Disease / Protein	ABC relationships of cells, Substances, diseases and organisms	Not applicable	MEDLINE titles and abstracts	Context-based and context-assignment-based models	Generation of new hypotheses of relations between cells, drugs, diseases and organisms	22
Association between autism and calcineurin	Semantic	Disease / Protein	ABC relationships based on rare terms linked with substances and phenomenons	Not applicable	MEDLINE full texts	RaJoLink method (semi-automated suggestions of candidates for starting and target concepts)	Relationships between biomedical concepts in disconnected articles	30
Association between cancer and antipsychotic drugs based on genes, brain-derived neurotrophic factor and glucocorticoid receptor	Semantic	Disease / Substance	Substance→B→ Disease	Not applicable	MEDLINE citations	Semantic predications (SemRep)	Mechanisms under-lying antipsychotic drugs therapy used in the treatment of cancer	1

Discoveries	LBD Category	Starting and target concepts	Discovery pattern	Natural products	Major repository	Underlying method	Core interest	Ref.
Dimebon (Latrepirdine) and Pirlindol with a potential in multiple sclerosis	Semantic	Disease / Substance	Disease→Gene→ Substance	Not applicable	Clinical outcome search space platform (incl. MED-LINE citations)	Systems literature analysis (SLA)	Drug repurposing	2
Association between migraine and zinc or retinoic acid	Semantic	Disease / Substance	Disease→Protein→ Substance	M, V	MEDLINE citations annotated by MeSH terms	ChemoText database on drug-target-disease associations	Drug discovery (to generate a hypothesis set of chemicals important to drug research)	4
Association between the obesity paradox in critical care and diethylhexyl phthalates	Semantic	Disease / Substance	ABC relationships of different biomedical concepts	Not applicable	MEDLINE titles and abstracts	Semantic predications (SemRep, Semantic MEDLINE)	Study of poorly understood areas of biomedicine	5
Drugs influencing genes associated with Parkinson's disease	Semantic	Disease / Substance	Disease→Gene→ Substance	N, ND, M	MEDLINE citations and GEO repository (GSE8397)	Combination of microarray data and semantic predications	New hypothesis for drug therapies for Parkinson's disease	20
Associations between wogonin, genistein, fisetin and cancer cells	Semantic	Disease / Substance	Disease→Biological process→ Substance	N	MEDLINE abstracts	Multi-level knowledge emergence model	Discovery of novel relationships	21
Genes associated with both myocardial infarction and depressive disorder	Semantic	Gene / Gene	Disease→Gene→ Disease	Not applicable	MEDLINE titles and abstracts, MeSH terms	BITOLA (interactive biomedical discovery support system)	Candidate genes for the interaction between diseases	14

Discoveries	LBD Category	Starting and target concepts	Discovery pattern	Natural products	Major repository	Underlying method	Core interest	Ref.
Association between genes of the beta-amyloid precursor protein and glycoprotein reelin	Semantic	Gene / Gene	ABC relationships with ranking of the B terms according to their likely relevance	Not applicable	MEDLINE citations	ARROWSMITH two node search including a logistic regression model for estimating the probability of relevance for B terms	Identification of implicit links in two disparate sets of articles	46
Association between genotypes and phenotypes of prokaryotic species	Semantic	Gene / Phenotype	Comparison of species-word and species-gene vectors for pre-dicting binary relationships	Not applicable	MEDLINE abstracts	Systematic approach combining literature mining and comparative genome analysis	Exploration of genotype-phenotype associations (functional genomics)	23
Associations between the enzyme calpain and the post-synaptic density	Semantic	Protein / Biological process	ABC relationships with ranking of the B terms according to their likely relevance	Not applicable	MEDLINE citations	ARROWSMITH two node search including a logistic regression model for estimating the probability of relevance for B terms	Identification of implicit links in two disparate sets of articles	46
Associations between Docetaxel and IL-1	Semantic	Protein / Substance	Protein→Biological process→ Substance	ND	MEDLINE abstracts	Multi-level knowledge emergence model	Discovery of novel relationships	21
Identification of compounds active against targets in the epidermal growth factor receptor (ErbB) signaling pathway with rel. to disease	Semantic	Protein / Substance	Substance→Gene→ Disease	Not applicable	MEDLINE citations SemMedDB	Semantic predication	Drug repositioning	34
Association between metabotropic glutamate receptors and Lewy bodies	Semantic	Receptor / Disease	ABC relationships with ranking of the B terms acc. to their relevance	Not applicable	MEDLINE citations	ARROWSMITH two node search including a logistic regression model	Identification of implicit links in two disparate sets of articles	46

Discoveries	LBD Category	Starting and target concepts	Discovery pattern	Natural products	Major repository	Underlying method	Core interest	Ref.
Identification of chemical matter for a dopamine receptor D_2 drug discovery program	Semantic	Receptor / Substance	Search query based on entities of interest contained in a triple store	Not defined[c]	Biomedical databases in Open PHACTS	Comprehensive integration of multiple data sources by using the platform Open Pharmacological Concepts Triple Store (openPHACTS)	Novel hypothesis in the process of drug discovery	34
Association between various drugs and multiple myeloma	Semantic	Substance / Disease	Substance→B→ Disease	N	MEDLINE citations	Predication-based Semantic Indexing (PSI)	Discovery of therap. useful connections	12
Association between statins and prostate cancer	Semantic	Substance / Disease	Substance→Protein→ Disease	Not defined[b]	MEDLINE abstracts or full texts	Advanced text mining based on information retrieval, natural language processing	Novel research hypothesis in biomedicine	24
Drugs with a potential in asthma, coronary heart disease, dermatitis, heart failure, hyper-tensive disease, ischemia, renal tubular disorder and tetanus	Semantic	Substance / Disease	Substance→Gene→ Disease	N, ND	MEDLINE citations, SemMedDB	Semantic predication	Drug repositioning	33
Association between Thalidomide and acute pancreatitis, chronic hepatitis C, *Helicobacter pylori*-induced gastritis and myasthenia gravis	Semantic	Substance / Disease	ABC relationships of the starting concept Thalidomide	Not applicable	MEDLINE titles and abstracts	Concept-based discovery support tool applying advanced natural language processing	Drug repurposing	48
Twenty-one drugs with a potential in prostate cancer	Semantic	Substance / Disease	Substance→Gene→ Disease	N, ND, V	MEDLINE titles and abstracts	Semantic predications (SemRep, MEDLINE)	Drug repurposing	54

Discoveries	LBD Category	Starting and target concepts	Discovery pattern	Natural products	Major repository	Underlying method	Core interest	Ref.
Sixty-two unknown pairs of potentially interacting drugs in the medication lists of 22 patients	Semantic	Substance / Substance	Substance→Gene→ Substance or Substance→Gene→ Biological function←Gene← Substance	Not defined[d]	MEDLINE titles and abstracts	Semantic predications (SemRep, Semantic MEDLINE)	Drug-drug interactions	55
Interactions between cancer drugs and Vitamin E; Interactions between glucosamine or melatonin and Docetaxel	Semantic	Substance / Substance	Substance→Gene→ Substance	ND, V	MEDLINE titles and abstracts	Semantic predications (SemRep, Semantic MEDLINE)	Interactions between cancer drugs and dietary supplements	57

Footnotes a to b: In cases indicated as "Not defined", we were not able to establish, whether the concepts mentioned in the respective study included natural products, because the information available is unspecific (i), codified (ii), or consisted of extensive lists of concepts (iii), the elucidation of which is outside of the scope and possibilities of this study: a) Chen et al. (2012) – the identities of the antipsychotic drugs are only provided by their Pubmed ID; b) Korhonen et al. (2015) – the term "statins" can refer to natural products, natural product derivatives or synthetic products; c) Ratnam et al. (2014) – the identities of the organic compounds active against DRD2 are only provided by their Pubmed ID; d), Rui Zhang et al. (2014) – the list of unknown drug-drug interactions consists of 62 cases; e) Yuji Zhang et al. (2014) – the sub-network associated with Alzheimer's disease and nervous system disorders consists of 51 drugs.

Table columns

Discoveries**:** A discovery is defined as a predicted relationship (i.e. hypothetical relationship) between two or more concepts and is represented by the concept association cited. Discoveries with identical semantic types of starting and target concepts reported in the same publication were pooled together and counted as one use case of LBD. Re-discoveries of previously published literature based discoveries also mentioned in the publications were not considered.

Starting and target concepts**:** The starting concept (A term in the ABC model of the discovery approach) and the target concept (C term) of the discoveries are elucidated and classed into semantic categories (e.g. disease, substance (incl. drug), gene, protein) based on information in the respective study or by using the resources listed below. In many cases (e.g. closed discoveries), starting and target concepts cannot be distinguished. Therefore, this categorisation follows a conceptual perspective with the principal aim of understanding the semantic types of the key concepts involved in the discoveries.

Discovery pattern: The principle discovery patterns of all studies listed follow the ABC model (A=starting concept, B=intermediate concept(s), C=target concept). With the exception of multidimensional pathways in association networks or concept spaces, discovery pathways are displayed, whenever possible, as A→B→C relationships of the related concepts (heterogeneous concepts of intermediate terms are indicated as "B").

Natural product**:** The presence of natural products among the concepts involved in the discoveries is indicated: N=Natural product from plants, microorganisms and higher fungi, including secondary metabolites and small molecules as well as complex mixtures such as herbal substances or extracts); ND=Natural product derivative (derivatives of the above group of natural products including semi-synthetic compounds); A=Animal venom; M=Mineral; V=Vitamin. The individual natural products are listed in **Supplementary Table 2**. If no natural products were present among the concepts of the discovery, this is indicated with "Not applicable". The definition of natural products applied essentially follows the definition of the Natural Centre for Complementary and Integrative Health (NCCIH) of the National Health Institute (NIH) (U.S. Department of Health and Human Services) (https://www.nccih.nih.gov/).

Major repository**:** Major literature resource used for establishing the knowledge database linked with the respective discovery. Concept databases (e.g. ontologies and dictionaries) potentially used in the study are listed only in cases where they represented the major source of information.

Underlying method**:** Principal approach of the method used in the study or, if appropriate, name of the respective LBD system.

Core interest**:** As far as possible the terminology used in the respective study was adopted. Therefore, some of the descriptions may be synonymous.

LBD category: The core LBD methodology used in the respective study either as Co-occurrence, Semantic, or Graph-base.

Ref.: Reference mentioning the respective discovery/-ies (Reference list, see at the end of this document).

Resources used for the classification of the concepts mentioned in the discoveries in Supplementary Table 1:

Newman, D. J.; Cragg, G. M. Natural Products as Sources of New Drugs over the Nearly Four Decades from 01/1981 to 09/2019. J. Nat. Prod. 2020, 83 (3), 770–803.

Patridge, E.; Gareiss, P.; Kinch, M. S.; Hoyer, D. An Analysis of FDA-Approved Drugs: Natural Products and Their Derivatives. Drug Discov. Today 2016, *21* (2), 204–207.

PubChem (https://pubchem.ncbi.nlm.nih.gov/), Medical subject headings (https://www.nlm.nih.gov/mesh/meshhome.html), ChemIDplus (https://chem.nlm.nih.gov/ chemidplus/) of the United States National Library of Medicine.

RÖMPP online, Lexicon of pharmaceuticals and natural products (https://roempp.thieme.de/). Böckler, F., Dill, B., Dingerdissen, U., Eisenbrand, G., Faupel, F., Fugmann, B., Gamse, T., Matissek, R., Pohnert, G., Sprenger, G. (Eds). Thieme: Stuttgart, 2020.

Supplementary Table 2. Literature-based discoveries involving natural products (The table is sorted according to references).

**Discoveries**	**Natural product relevant concept**	**Identity of the concept**	**Natural product category**	**Natural source**	**Taxonomic information about the natural source**	**Ref.**
Azithromycin with a potential in cystic fibrosis	Azithromycin	Antibiotic	ND	Microorganism	*Saccharopolyspora erythraea* (Waksman, 1923) Labeda, 1987, *Streptomyces griseoplanus* Backus et al., 1957, and other bacteria	3
Genistein with a potential in cystic fibrosis	Genistein	Isoflavone	N	Plant	*Genista tinctoria* L., *Glycine max* (L.) Merr. [Fabaceae] and various other taxa	3
Mannitol with a potential in cystic fibrosis	Mannitol	Sugar alcohol	N	Plant, Fungus	*Fraxinus ornus* L. (manna) [Oleaceae], *Laminaria cloustoni* Edmonston and other seaweed	3
Curcumin with a potential in cystic fibrosis	Curcumin	Curcuminoid	N	Plant	*Curcuma longa* L. (root) [Zingiberaceae]	3
Rifampin with a potential in cystic fibrosis	Rifampin	Antibiotic	ND	Microorganism	*Amycolatopsis rifamycinica* Bala et al., 2004 (syn. *Streptomyces mediterranei)*	3
Lithium with a potential in cystic fibrosis	Lithium	Mineral	M	Mineral	Various sources	3
Forskolin with a potential in cystic fibrosis	Forskolin	Diterpene	N	Plant	*Plectranthus hadiensis* (Forssk.) Schweinf. ex Sprenger (syn. *Coleus forskohlii*) [Lamiaceae]	3
Caffeine with a potential in cystic fibrosis	Caffeine	Alkaloid	N	Plant	*Coffea arabica* L., *C. canephora* Pierre ex A.Froehner [Rubiaceae], *Camellia sinensis* (L.) Kuntze [Theaceae], and other plants	3
Capsaicin with a potential in migraine	Capsaicin	Alkaloid	N	Plant	*Capsicum* spp. (fruit) [Solanaceae]	3
Mycophenolate mofetil with a potential in psoriasis	Mycophenolate mofetil	Antibiotic	ND	Fungus	*Penicillium brevicompactum* Dierckx	3

**Discoveries**	**Natural product relevant concept**	**Identity of the concept**	**Natural product category**	**Natural source**	**Taxonomic information about the natural source**	**Ref.**
Paclitaxel with a potential in psoriasis	Paclitaxel	Diterpene	N	Plant	*Taxus brevifolia* Nutt. (bark), *T. baccata* L. (leaf) [Taxaceae] and other plants	3
Capsaicin with a potential in psoriasis	Capsaicin	Alkaloid	N	Plant	*Capsicum* spp. (fruit) [Solanaceae]	3
Rifampin with a potential in psoriasis	Rifampin	Antibiotic	ND	Microorganism	*Amycolatopsis rifamycinica* Bala et al., 2004 (syn. *Streptomyces mediterranei)*	3
Erythromycin with a potential in psoriasis	Erythromycin	Antibiotic	ND	Microorganism	*Streptomyces antibioticus* (Waksman & Woodruff, 1941) Waksman & Henrici, 1948	3
Association between migraine and zinc	Zinc	Mineral	M	Mineral	Various sources	4
Association between migraine and retinoic acid	Retinoic acid	Vitamin	V	Animal, plant	Various sources	4
Association between cobratoxin and the acetylcholine receptor	Cobratoxin	Venom	A	Animal	Snakes of the genus *Naja*	11
Association between cobratoxin and the nicotinic acetylcholinesterase receptor	Cobratoxin	Venom	A	Animal	Snakes of the genus *Naja*	11
Association between cobratoxin and the plant neurotoxin erythroidine (based on similar actions)	Cobratoxin / Erythroidine	Venom / Alkaloid	A / N	Animal / Plant	Snakes of the genus *Naja* / *Erythrina* spp. [Fabaceae]	11
Association between cobratoxin and Bungarus toxins	Cobratoxin / BuTx, BTx, BgTx (Bungarus toxin)	Venom / Venom	A / A	Animal / Animal	Snakes of the genus *Naja /* *Bungarus multicinctus* (Blyth, 1861) and other snakes of the genus *Bungarus*	11
Association between cobratoxin and conotoxin	Cobratoxin / Conotoxin	Venom / Venom	A / A	Animal / Animal	Snakes of the genus *Naja* / Marine snails of the genus *Conus*	11

**Discoveries**	**Natural product relevant concept**	**Identity of the concept**	**Natural product category**	**Natural source**	**Taxonomic information about the natural source**	**Ref.**
Association between cobratoxin and dendrotoxin	Cobratoxin / Dendrotoxin	Venom / Venom	A / A	Animal / Animal	Snakes of the genus *Naja* / Snakes of the genus *Dendroapsis*	11
Association between cobratoxin and Ls-III neurotoxin	Cobratoxin / Laticauda semifasciata III neurotoxin	Venom / Venom	A / A	Animal / Animal	Snakes of the genus *Naja /* Marine snake *Laticauda semifasciata* Reinwardt, 1837	11
Association between cobratoxin and suberyldicholine	Cobratoxin	Venom	A	Animal	Snakes of the genus *Naja*	11
Association between cobratoxin and the maleimid scafold	Cobratoxin	Venom	A	Animal	Snakes of the genus *Naja*	11
Association between cobratoxin and electric fish muscle fibres	Cobratoxin	Venom	A	Animal	Snakes of the genus *Naja*	11
Animal taxa containing in their venom cobratoxin	Cobratoxin	Venom	A	Animal	Snakes of the genus *Naja*	11
Association between convulxin and the antiaggregatory effect	Convulxin	Venom	A	Animal	Rattle snake *Crotalus durissus* ssp. *terrificus* Laurenti, 1768	11
Association between convulxin and the aggregability of blood platelets	Convulxin	Venom	A	Animal	Rattle snake *Crotalus durissus* ssp. *terrificus* Laurenti, 1768	11
Association between convulxin and citrated compounds	Convulxin	Venom	A	Animal	Rattle snake *Crotalus durissus* ssp. *terrificus* Laurenti, 1768	11
Association between convulxin and thrombospondin	Convulxin	Venom	A	Animal	Rattle snake *Crotalus durissus* ssp. *terrificus* Laurenti, 1768	11

**Discoveries**	**Natural product relevant concept**	**Identity of the concept**	**Natural product category**	**Natural source**	**Taxonomic information about the natural source**	**Ref.**
Association between convulxin and glycoproteins ib, iib, iiia, v	Convulxin	Venom	A	Animal	Rattle snake *Crotalus durissus* ssp. *terrificus* Laurenti, 1768	11
Association between convulxin and the anticoagulants hirudin and apyrase	Convulxin	Venom	A	Animal	Rattle snake *Crotalus durissus* ssp. *terrificus* Laurenti, 1768	11
Association between convulxin and ristocetin	Convulxin / Ristocetin	Venom / Antibiotic	A / N	Animal / Microorganism	Rattle snake *Crotalus durissus* ssp. *terrificus* Laurenti, 1768 / *Amycolatopsis lurida* (Lechevalier et al., 1986) Stackebrandt et al., 2004 and other taxa of the order Actinomycetales	11
Association between convulxin and and platelet-rich plasma	Convulxin	Venom	A	Animal	Rattle snake *Crotalus durissus* ssp. *terrificus* Laurenti, 1768	11
Animal taxa containing in their venom convulxin or related venoms	Convulxin	Venom	A	Animal	Rattle snake *Crotalus durissus* ssp. *terrificus* Laurenti, 1768	11
Association between curcumin and multiple myeloma	Curcumin	Curcuminoid	N	Plant	*Curcuma longa* L. (root) [Zingiberaceae]	12
Association between adriamycin and multiple myeloma	Adriamycin (Doxorubicin)	Antibiotic	N	Microorganism	*Streptomyces peucetius* Grein et al., 1963	12
Association between genistein and multiple myeloma	Genistein	Isoflavone	N	Plant	*Genista tinctoria* L., *Glycine max* (L.) Merr. [Fabaceae] and various other taxa	12
Association between quercetin and multiple myeloma	Quercetin	Flavonol	N	Plant	*Quercus* ssp. (bark) [Fagaceae] and other taxa	12
Association between zinc and multiple myeloma	Zinc	Mineral	M	Mineral	Various sources	12

Discoveries	Natural product relevant concept	Identity of the concept	Natural product category	Natural source	Taxonomic information about the natural source	Ref.
Association between Damnacanthal and cell proliferation	Damnacanthal	Anthraquinone	N	Plant	*Morinda lucida* Benth., *M. citrifolia* L. (root or bark) [Rubiaceae]	15
Association between Dephostatin and cell proliferation	Dephostatin	Statin	N	Microorganism	*Streptomyces* sp.	15
Influence of Paclitaxel on genes associated with Parkinson's disease	Paclitaxel	Diterpene	N, ND	Plant	*Taxus brevifolia* Nutt. (bark), *T. baccata* L. (leaf) [Taxaceae]	20
Influence of quercetin on genes associated with Parkinson's disease	Quercetin	Flavonol	N	Plant	*Quercus* ssp. (bark) [Fagaceae] and various other taxa	20
Influence of iron on genes associated with Parkinson's disease	Iron	Mineral	M	Mineral	Various sources	20
Association between wogonin and cancer cells	Wogonin	Flavone	N	Plant	*Scutellaria baicalensis* Georgi [Lamiaceae]	21
Association between genistein and cancer cells	Genistein	Isoflavone	N	Plant	*Genista tinctoria* L., *Glycine max* (L.) Merr. [Fabaceae] and various other taxa	21
Association between fisetin and cancer cells	Fisetin	Flavonol	N	Plant	*Cotinus coggygria* Scop. [Anacardiaceae], and other Anacardiaceae or Fabaceae	21
Association between fisetin and malignant T cells	Fisetin	Flavonol	N	Plant	*Cotinus coggygria* Scop. [Anacardiaceae], and other Anacardiaceae or Fabaceae	21
Association between Docetaxel and IL-1	Docetaxel	Diterpene	ND	Plant	*Taxus brevifolia* Nutt. (bark), *T. baccata* L. (leaf) [Taxaceae]	21
Quinidine and its potential in Alzheimer's disease	Quinidine	Quinoline alkaloid	N	Plant	*Cinchona officinalis* L., *C. calisaya* Wedd., or *C. pubescens* Vahl (bark) [Rubiaceae]	25

Discoveries	Natural product relevant concept	Identity of the concept	Natural product category	Natural source	Taxonomic information about the natural source	Ref.
Scopolamine with a potential in Alzheimer’s disease	Scopolamine	Tropane alkaloid	N	Plant	*Datura* spp. and other Solanaceae	26
Bromocriptine with a potential in Alzheimer’s disease	Bromocriptine	Ergot alkaloid	ND	Fungus	*Claviceps purpurea* (Fr.) Tul., and other ergot fungi or ergot alkaloids containing plants	26
Benzatropine with a potential in Alzheimer’s disease	Benzatropine	Tropane alkaloid	ND	Plant	*Atropa belladonna* L. and other Solanaceae	26
Association between arsenic and autotaxin and other genes	Arsenic	Mineral	M	Mineral	Various sources	31
Tretinoin with a potential in asthma	Tretinoin (Airol)	Retinoid	ND	Plant	Various sources	33
Cyclosporine with a potential in asthma	Cyclosporine	Antibiotic	N	Fungus	*Tolypocladium inflatum* W.Gams, and other strains of soil fungi	33
Ezetimibe with a potential in coronary heart disease	Ezetimibe	Azetidine	ND	Plant	*Convallaria majalis* L. [Asparagaceae]	33
Association between magnesium deficiency and neurologic disease	Magnesium	Mineral	M	Mineral	Various sources	35
Association between schizophrenia and calcium independent phospholipase A2 based on Vitamin E	Vitamin E	Vitamin	V	Animal, plant	Various sources	38
Association between curcumin and retinal diseases through influence on genes or gene products	Curcumin	Curcuminoid	N	Plant	*Curcuma longa* L. (root) [Zingiberaceae]	40
Association between curcumin and Crohn’s disease through influence on genes or gene products	Curcumin	Curcuminoid	N	Plant	*Curcuma longa* L. (root) [Zingiberaceae]	40

**Discoveries**	**Natural product relevant concept**	**Identity of the concept**	**Natural product category**	**Natural source**	**Taxonomic information about the natural source**	**Ref.**
Association between curcumin and disorders related to the spinal cord through influence on genes or gene products	Curcumin	Curcuminoid	N	Plant	*Curcuma longa* L. (root) [Zingiberaceae]	40
Association between fish oil and Raynaud's disease through the influence on blood circulation	Fish oil	Fatty acid mixture	N	Animal (fish), plant	Fatty fish or omega-3 fatty acids producing microalgae	41
Association between magnesium and migraine through various physiological and clinical factors	Magnesium	Mineral	M	Mineral	Various sources	42
Ileum used in experiments to test capsaicin's effect on muscle contraction	Capsaicin	Alkaloid	N	Plant	*Capsicum* spp. (fruit) [Solanaceae]	51
Association between capsaicin and atropine	Atropine / Capsaicin	Alkaloid / Alkaloid	N / N	Plant / Plant	*Atropa belladonna* L. and other Solanaceae / *Capsicum* spp. (fruit) [Solanaceae]	51
Association between capsaicin and tetrodotoxin	Tetrodotoxin / Capsaicin	Alkaloid / Alkaloid	N / N	Animal (fish) / Plant	*Takifugu rubripes* (Temminck & Schlegel, 1850) (syn. *Spheroides rubripes*) / *Capsicum* spp. (fruit) [Solanaceae]	51
Bryostatin 1 with a potential in prostate cancer	Bryostatin 1	Polyketide	N	Animal (bryozoa, marine)	*Bugula neritina* (Linnaeus, 1758)	54
Curcumin with a potential in prostate cancer	Curcumin	Curcuminoid	N	Plant	*Curcuma longa* L. (root) [Zingiberaceae]	54
Catechin with a potential in prostate cancer	Catechin	Polyphenol	N	Plant	Widely distributed in vascular plants, e.g. *Camellia sinensis* (L.) Kuntze [Theaceae]	54

**Discoveries**	**Natural product relevant concept**	**Identity of the concept**	**Natural product category**	**Natural source**	**Taxonomic information about the natural source**	**Ref.**
Lovastatin with a potential in prostate cancer	Lovastatin	Polyketide	N	Fungus	*Aspergillus terreus* Thom, other Ascomycota such as *Monascus* spp. or Basidiomycota such as *Pleurotus* spp.	54
Paclitaxel with a potential in prostate cancer	Paclitaxel	Diterpene	N	Plant	*Taxus brevifolia* Nutt. (bark), *T. baccata* L. (leaf) [Taxaceae]	54
Metformin with a potential in prostate cancer	Metformin	Biguanidine	ND	Plant	*Galega officinalis* L. [Fabaceae]	54
Nicotinic acid with a potential in prostate cancer	Nicotinic acid (niacin, form of vitamin B3)	Vitamin	V	Animal, plant	Various sources	54
Association between the chemotherapy side effects of Doxorubicin and Dobutamine used in heart diseases	Adriamycin (Doxorubicin)	Antibiotic	N	Microorganism	*Streptomyces peucetius* Grein et al., 1963	56
Interaction (known) between Echinacea and Cyclophosphamide	Echinacea	Botanical	N	Plant	*Echinacea purpurea* (L.) Moench (herb) [Asteraceae], or other kinds of "Echinacea" (unspecific term)	57
Interaction (known) between Echinacea and Docetaxel	Echinacea / Docetaxel	Botanical / Diterpene	N / ND	Plant / Plant	*Echinacea purpurea* (L.) Moench (herb) [Asteraceae], or other kinds of "Echinacea" (unspecific term) / *Taxus brevifolia* Nutt. (bark), *T. baccata* L. (leaf) [Taxaceae]	57
Interaction (known) between Echinacea and Everolimus	Echinacea / Everolimus	Botanical / Macrolide	N / ND	Plant / Microorganism	*Echinacea purpurea* (L.) Moench (herb) [Asteraceae], or other kinds of "Echinacea" (unspecific term) / *Streptomyces hygroscopicus* (Jensen, 1931) Waksman & Henrici, 1948	57

**Discoveries**	**Natural product relevant concept**	**Identity of the concept**	**Natural product category**	**Natural source**	**Taxonomic information about the natural source**	**Ref.**
Interaction (unknown) between Echinacea and Exemestane	Echinacea	Botanical	N	Plant	*Echinacea purpurea* (L.) Moench (herb) [Asteraceae], or other kinds of "Echinacea" (unspecific term)	57
Interaction (known) between Echinacea and Fluorouracil	Echinacea	Botanical	N	Plant	*Echinacea purpurea* (L.) Moench (herb) [Asteraceae], or other kinds of "Echinacea" (unspecific term)	57
Interaction (unknown) between Echinacea and Toremifene	Echinacea	Botanical	N	Plant	*Echinacea purpurea* (L.) Moench (herb) [Asteraceae], or other kinds of "Echinacea" (unspecific term)	57
Interaction (unknown) between Ginseng and Docetaxel	Ginseng / Docetaxel	Botanical / Diterpene	N / ND	Plant / Plant	*Panax ginseng* C.A.Meyer (root) [Araliaceae], or other kinds of "ginseng" (unspecific term) / *Taxus brevifolia* Nutt. (bark), *T. baccata* L. (leaf) [Taxaceae]	57
Interaction (unknown) between Grape seed extract and Docetaxel	Grape seed extract / Docetaxel	Botanical / Diterpene	N / ND	Plant / Plant	*Vitis vinifera* L. (seed) [Vitaceae] / *Taxus brevifolia* Nutt. (bark), *T. baccata* L. (leaf) [Taxaceae]	57
Interaction (known) between Kava preparation and Docetaxel	Kava preparation / Docetaxel	Botanical / Diterpene	N / ND	Plant / Plant	*Piper methysticum* G.Forst. (root) [Piperaceae] / *Taxus brevifolia* Nutt. (bark), *T. baccata* L. (leaf) [Taxaceae]	57
Interaction (known) between Ginseng and Ginkgo biloba extract	Ginseng / Ginkgo biloba extract	Botanical / Botanical	N / N	Plant / Plant	*Panax ginseng* C.A.Meyer [Araliaceae], or other sources for different qualities of "ginseng" / *Ginkgo biloba* L. (leaf) [Ginkgoaceae]	57
Interaction (unknown) between Vitamin E and Cyclophosphamide	Vitamin E	Vitamin	V	Animal, plant	Various sources	57
Interaction (unknown) between Vitamin E and Prednisone	Vitamin E	Vitamin	V	Animal, plant	Various sources	57

**Discoveries**	**Natural product relevant concept**	**Identity of the concept**	**Natural product category**	**Natural source**	**Taxonomic information about the natural source**	**Ref.**
Interaction (unknown) between melatonin and Docetaxel	Docetaxel	Diterpene	ND	Plant	*Taxus brevifolia* Nutt. (bark), *T. baccata* L. (leaf) [Taxaceae]	57
Interaction (known) between glucosamine and Docetaxel	Glucosamine / Docetaxel	Mono-saccharide / Diterpene	N / ND	Animal, Fungi / Plant	Various sources including shellfish exoskeletons as one major commercial source / *Taxus brevifolia* Nutt. (bark), *T. baccata* L. (leaf) [Taxaceae]	57

Table columns

Discoveries: A discovery is defined as a predicted relationship between two or more concepts and is represented by the concept association cited.

Natural product relevant concept: Concepts mentioned in the respective discovery that are classes as natural products. The definition of natural products applied essentially follows the definition of the Natural Centre for Complementary and Integrative Health (NCCIH) of the National Health Institute (NIH) (U.S. Department of Health and Human Services) (https://www.nccih.nih.gov/).

Identity of the concept: Classification of the natural product related concepts into semantic categories. Substances were classed into compound classes (based on the literature used for the compilation of the taxonomic information, see below)

Natural product category: N=Natural product from plants, microorganisms and higher fungi, including secondary metabolites and small molecules as well as complex mixtures such as herbal substances or extracts); ND=Natural product derivative (derivatives of the above group of natural products including semi-synthetic compounds); A=Animal venom; M=Mineral; V=Vitamin.

Natural source: The source of the natural product is indicated by using one of the following categories: plant, animal, fungus, microorganism, mineral.

Taxonomic information about the source: The taxonomic identity of the natural source is elucidated using the resources listed below. Priority is given to the source from which the natural product originally was discovered. If appropriate, additional important natural sources are listed. The taxonomic information stated in the resources was verified using Kew's Medicinal Plant Names Services (https://mpns.science.kew.org/mpns-portal/) or Plants of the World online (http://www.plantsof theworldonline.org/) for plants, and Global Biodiversity Information Facility (GBIF) (https://www.gbif.org/) for all other organisms. For plants, information about the relevant plant part (if available) as well as the family is indicated, e.g. *Taxus brevifolia* Nutt. (bark), [Taxaceae].

Ref.: Reference mentioning the respective discovery/-ies (Reference list, see at the end of this document).

Resources used for the elucidation of the source of the natural product mentioned in the discoveries in Supplementary Table 2:

Dictionary of Antibiotics and Related Substances; Bycroft, B. W., Payne, D. J. (Eds). CRC Press: Boca Raton, 2013.

HagerROM 2009: Hagers Enzyklopädie der Arzneistoffe und Drogen; Blaschek, W., Hilgenfeldt, U., Holzgrabe, U., Reichling, J., Ruth, P., Schulz, V. (Eds). Springer: Berlin, 2009.

Newman, D. J.; Cragg, G. M. Natural Products as Sources of New Drugs over the Nearly Four Decades from 01/1981 to 09/2019. J. Nat. Prod. 2020, 83 (3), 770–803.

Patridge, E.; Gareiss, P.; Kinch, M. S.; Hoyer, D. An Analysis of FDA-Approved Drugs: Natural Products and Their Derivatives. Drug Discov. Today 2016, *21* (2), 204–207.

PubChem (https://pubchem.ncbi.nlm.nih.gov/), Medical subject headings (https://www.nlm.nih.gov/mesh/meshhome.html), ChemIDplus (https://chem.nlm.nih.gov/ chemidplus/) of the United States National Library of Medicine.

RÖMPP online, Lexicon of pharmaceuticals and natural products (https://roempp.thieme.de/). Böckler, F., Dill, B., Dingerdissen, U., Eisenbrand, G., Faupel, F., Fugmann, B., Gamse, T., Matissek, R., Pohnert, G., Sprenger, G. (Eds). Thieme: Stuttgart, 2020.

Vardanian, R.; Hruby, V. Synthesis of Best-Seller Drugs. Academic Press: London, 2016.

Reference list of the studies mentioning the use cases of literature-based discovery (LBD) cited in the Supplementary Tables 1 and 2

1. Ahlers, C. B., Hristovski, D., Kilicoglu, H. & Rindflesch, T. C. Using the literature-based discovery paradigm to investigate drug mechanisms. *AMIA Annu. Symp. Proc.* 2007, 6–10 (2007).
2. Andronis, C., Sharma, A., Virvilis, V., Deftereos, S. & Persidis, A. Literature mining, ontologies and information visualization for drug repurposing. *Brief. Bioinform.* 12, 357–368 (2011).
3. Baker, N. C. (2010). *Methods in Literature-based Drug Discovery* (Doctoral thesis). The University of North Carolina at Chapel Hill. ProQuest Dissertations Publishing.
4. Baker, N. C. & Hemminger, B. M. Mining connections between chemicals, proteins, and diseases extracted from Medline annotations. *J. Biomed. Inform.* 43, 510–519 (2010).
5. Cairelli, M. J., Miller, C. M., Fiszman, M., Workman, T. E. & Rindflesch, T. C. Semantic MEDLINE for discovery browsing: using semantic predications and the literature-based discovery paradigm to elucidate a mechanism for the obesity paradox. *AMIA Annu. Symp. Proc.* 2013, 164–173 (2013).
6. Capuzzi, S. J. *et al.* Chemotext: A Publicly Available Web Server for Mining Drug-Target-Disease Relationships in PubMed. *J. Chem. Inf. Model.* 58, 212–218 (2018).
7. Chen, H. & Sharp, B. M. Content-rich biological network constructed by mining PubMed abstracts. *BMC Bioinformatics* 5, 147 (2004).
8. Chen, X., Liu, M. X. & Yan, G. Y. Drug-target interaction prediction by random walk on the heterogeneous network. *Mol. Biosyst.* 8, 1970–1978 (2012).
9. Cockell, S. J. *et al.* An integrated dataset for in silico drug discovery. *J. Integr. Bioinform.* 7, (2010).
10. Cohen, T., Whitfield, G. K., Schvaneveldt, R. W., Mukund, K. & Rindflesch, T. EpiphaNet: An Interactive Tool to Support Biomedical Discoveries. *J. Biomed. Discov. Collab.* 5, 21–49 (2010).
11. Cohen, T., Schvaneveldt, R. & Widdows, D. Reflective Random Indexing and indirect inference: A scalable method for discovery of implicit connections. *J. Biomed. Inform.* 43, 240–256 (2010).
12. Cohen, T., Widdows, D., Schvaneveldt, R. W., Davies, P. & Rindflesch, T. C. Discovering discovery patterns with predication-based Semantic Indexing. *J. Biomed. Inform.* 45, 1049–1065 (2012).
13. Crichton, G. K. O. (2019). *Improving Automated Literature-based Discovery with Neural Networks: Neural biomedical Named Entity Recognition, Link Prediction and Discovery* (Doctoral thesis). University of Cambridge.
14. Dai, Z. *et al.* Using literature-based discovery to identify candidate genes for the interaction between myocardial infarction and depression. *BMC Med. Genet.* 20, 104 (2019).
15. Frijters, R. *et al.* Literature Mining for the Discovery of Hidden Connections between Drugs, Genes and Diseases. *PLoS Comput. Biol.* 6, e1000943 (2010).
16. Gramatica, R. *et al.* Graph Theory Enables Drug Repurposing – How a Mathematical Model Can Drive the Discovery of Hidden Mechanisms of Action. *PLoS One* 9, e84912 (2014).
17. Hristovski, D., Peterlin, B., Mitchell, J. A. & Humphrey, S. M. Improving Literature Based Discovery Support by Genetic Knowledge Integration. *Studies in Health Technology and Informatics* 68–73 (2003).
18. Hristovski, D., Peterlin, B., Mitchell, J. A. & Humphrey, S. M. Using literature-based discovery to identify disease candidate genes. *Int. J. Med. Inform.* 74, 289–298 (2005).
19. Hristovski, D., Friedman, C., Rindflesch, T. C. & Peterlin, B. Exploiting semantic relations for literature-based discovery. *AMIA Annu. Symp. Proc.* 2006, 349–353 (2006).

20. Hristovski, D., Kastrin, A., Peterlin, B. & Rindflesch, T. C. Combining Semantic Relations and DNA Microarray Data for Novel Hypotheses Generation. *Linking Literature, Information, and Knowledge for Biology* 53–61 (2010).

21. Ijaz, A. Z., Song, M. & Lee, D. MKEM: a Multi-level Knowledge Emergence Model for mining undiscovered public knowledge. *BMC Bioinformatics* 11 Suppl 2, S3 (2010).

22. Kim, Y. H. & Song, M. A context-based ABC model for literature-based discovery. *PLoS One* 14, e0215313 (2019).

23. Korbel, J. O. *et al.* Systematic association of genes to phenotypes by genome and literature mining. *PLoS Biol.* 3, e134 (2005).

24. Korhonen A. et al. (2015). Improving Literature-Based Discovery with Advanced Text Mining. In: DI Serio C., Liò P., Nonis A., Tagliaferri R. (eds) Computational Intelligence Methods for Bioinformatics and Biostatistics. CIBB 2014. *Lecture Notes in Computer Science*, vol 8623.

25. Li, J., Zhu, X. & Chen, J. Y. Building disease-specific drug-protein connectivity maps from molecular interaction networks and PubMed abstracts. *PLoS Comput. Biol.* 5, e1000450 (2009).

26. Liu, H., Song, Y., Guan, J., Luo, L. & Zhuang, Z. Inferring new indications for approved drugs via random walk on drug-disease heterogenous networks. *BMC Bioinformatics* 17, (2016).

27. Miller, C. M. *et al.* A closed literature-based discovery technique finds a mechanistic link between hypogonadism and diminished sleep quality in aging men. *Sleep* 35, 279–285 (2012).

28. Narayanasamy, V., Mukhopadhyay, S., Palakal, M. & Potter, D. A. TransMiner: Mining transitive associations among biological objects from text. *J. Biomed. Sci.* 11, 864–873 (2004).

29. Ozgür, A., Xiang, Z., Radev, D. R. & He, Y. Literature-based discovery of IFN-gamma and vaccine-mediated gene interaction networks. *J. Biomed. Biotechnol.* 2010, 426479 (2010).

30. Petrič, I., Urbančič, T., Cestnik, B. & Macedoni-Lukšič, M. Literature mining method RaJoLink for uncovering relations between biomedical concepts. *J. Biomed. Inform.* (2009) doi:10.1016/j.jbi.2008.08.004.

31. Pyysalo, S. *et al.* LION LBD: a literature-based discovery system for cancer biology. *Bioinformatics* 35, 1553–1561 (2019).

32. Qu, X. A., Gudivada, R. C., Jegga, A. G., Neumann, E. K. & Aronow, B. J. Inferring novel disease indications for known drugs by semantically linking drug action and disease mechanism relationships. *BMC Bioinformatics* 10 Suppl 5, S4 (2009).

33. Rastegar-Mojarad, M., Elayavilli, R. K., Li, D., Prasad, R. & Liu, H. A new method for prioritizing drug repositioning candidates extracted by literature-based discovery. in *2015 IEEE International Conference on Bioinformatics and Biomedicine (BIBM)* 669–674 (IEEE, 2015).

34. Ratnam, J. *et al.* The application of the open pharmacological concepts triple store (open PHACTS) to support drug discovery research. *PLoS One* 9, e115460 (2014).

35. Smalheiser, N. R. & Swanson, D. R. Assessing a gap in the biomedical literature-magnesium-deficiency and neurologic disease. *Neurosci. Res. Commun.* 15, 1–9 (1994).

36. Smalheiser, N. R. & Swanson, D. R. Indomethacin and Alzheimer's disease. *Neurology* 46, 583 (1996).

37. Smalheiser, N. R. & Swanson, D. R. Linking estrogen to Alzheimer's disease: an informatics approach. *Neurology* 47, 809–810 (1996).

38. Smalheiser, N. R. & Swanson, D. R. Using ARROWSMITH: a computer-assisted approach to formulating and assessing scientific hypotheses. *Comput. Methods Programs Biomed.* 57, 149–153 (1998).

39. Song, M., Han, N.-G., Kim, Y.-H., Ding, Y. & Chambers, T. Discovering implicit entity relation with the gene-citation-gene network. *PLoS One* 8, e84639 (2013).

40. Srinivasan, P. & Libbus, B. Mining MEDLINE for implicit links between dietary substances and diseases. *Bioinformatics* 20, i290–i296 (2004).
41. Swanson, D. R. Fish Oil, Raynaud's Syndrome, and Undiscovered Public Knowledge. *Perspect. Biol. Med.* 30, 7–18 (1986).
42. Swanson, D. R. Migraine and Magnesium: Eleven Neglected Connections. *Perspect. Biol. Med.* 31, 526–557 (1988).
43. Swanson, D. R. Medical literature as a potential source of new knowledge. *Bull. Med. Libr. Assoc.* 78, 29–37 (1990).
44. Sybrandt, J., Shtutman, M. & Safro, I. MOLIERE: Automatic Biomedical Hypothesis Generation System. *KDD* 2017, 1633–1642 (2017).
45. Thaicharoen, S., Altman, T., Gardiner, K. & Cios, K. J. Discovering relational knowledge from two disjoint sets of literatures using inductive logic programming. in *2009 IEEE Symposium on Computational Intelligence and Data Mining, CIDM 2009 - Proceedings* (2009). doi:10.1109/CIDM.2009.4938661.
46. Torvik, V. I. & Smalheiser, N. R. A quantitative model for linking two disparate sets of articles in MEDLINE. *Bioinformatics* 23, 1658–1665 (2007).
47. van der Eijk, C. C., van Mulligen, E. M., Kors, J. A., Mons, B. & van den Berg, J. Constructing an associative concept space for literature-based discovery. *J. Am. Soc. Inf. Sci. Technol.* 55, 436–444 (2004).
48. Weeber, M. *et al.* Generating hypotheses by discovering implicit associations in the literature: a case report of a search for new potential therapeutic uses for thalidomide. *J. Am. Med. Inform. Assoc.* 10, 252–259 (2003).
49. Wilkowski, B. *et al.* Graph-based methods for discovery browsing with semantic predications. *AMIA Annu. Symp. Proc.* 2011, 1514–1523 (2011).
50. Wren, J. D., Bekeredjian, R., Stewart, J. A., Shohet, R. V. & Garner, H. R. Knowledge discovery by automated identification and ranking of implicit relationships. *Bioinformatics* 20, 389–398 (2004).
51. Wren, J. D. Extending the mutual information measure to rank inferred literature relationships. *BMC Bioinformatics* 5, 145 (2004).
52. Wren, J. D. & Garner, H. R. Data-Mining Analysis Suggests an Epigenetic Pathogenesis for Type 2 Diabetes. *J. Biomed. Biotechnol.* 2005, 104 (2005).
53. Yetisgen-Yildiz, M. & Pratt, W. Using statistical and knowledge-based approaches for literature-based discovery. *J. Biomed. Inform.* 39, 600–611 (2006).
54. Zhang, R. *et al.* Exploiting Literature-derived Knowledge and Semantics to Identify Potential Prostate Cancer Drugs. *Cancer Inform.* 13s1, CIN.S13889 (2014).
55. Zhang, R. *et al.* Using semantic predications to uncover drug–drug interactions in clinical data. *J. Biomed. Inform.* 49, 134–147 (2014).
56. Zhang, Y. *et al.* Network-based analysis reveals distinct association patterns in a semantic MEDLINE-based drug-disease-gene network. *J. Biomed. Semantics* 5, 33 (2014).
57. Zhang, R. *et al.* Mining Biomedical Literature to Explore Interactions between Cancer Drugs and Dietary Supplements. *AMIA Joint Summits on Translational Science proceedings. AMIA Joint Summits on Translational Science* 2015, 69–73 (2015).
