# Supplementary material for: Computational Literature-based Discovery for Natural Products Research: Current State and Future Prospects
Source: Front Bioinform. 2022 Mar 15;2:827207. doi: 10.3389/fbinf.2022.827207 (PMC9580913; doi:10.3389/fbinf.2022.827207)
Supplement: Supplementary file 2 [file DataSheet1.pdf]

Supplementary Material: List of search terms

Article title: Computational literature-based discovery for natural products research: current state and future prospects

Authors: Andreas Lardos, Ahmad Aghaebrahimian, Anna Koroleva, Julia Sidorova, Evelyn Wolfram, Maria Anisimova, Manuel Gil

List of search terms used for identifying studies on LBD in the biomedical domain

Resources used: PubMed, Google Scholar, Semantic Scholar, ScienceDirect

Period of search: 01/2020 to 05/2020

Search terms

literature based discovery

data-driven drug discovery

knowledge-led drug discovery

text-based discovery

literature-related discovery

knowledge discovery in databases (KDD)

knowledge discovery in text (KDT)

lbdd

lbd

automated LBD

drug discovery

pharmaceutical discovery

drug repurposing

drug repositioning

drug reprofiling

mining undiscovered public knowledge

hypothesis generation (HG) systems

hypothesis generation

high-throughput screening

high-throughput literature analysis

drug relabeling

drug target discovery

generating hypotheses

hypothesis discovery
